# The Importance of Respiratory Rate Monitoring: From Healthcare to Sport and Exercise

**DOI:** 10.3390/s20216396

**Published:** 2020-11-09

**Authors:** Andrea Nicolò, Carlo Massaroni, Emiliano Schena, Massimo Sacchetti

**Affiliations:** 1 Department of Movement, Human and Health Sciences, University of Rome “Foro Italico”, 00135 Rome, Italy; massimo.sacchetti@uniroma4.it; 2 Unit of Measurements and Biomedical Instrumentation, Department of Engineering, Università Campus Bio-Medico di Roma, Via Alvaro del Portillo, 21, 00128 Rome, Italy; c.massaroni@unicampus.it (C.M.); e.schena@unicampus.it (E.S.)

**Keywords:** respiratory frequency, respiratory monitoring, technologies, sensors, wearables, vital signs, stress, breathing control, measurement scenario, patient monitoring

## Abstract

Respiratory rate is a fundamental vital sign that is sensitive to different pathological conditions (e.g., adverse cardiac events, pneumonia, and clinical deterioration) and stressors, including emotional stress, cognitive load, heat, cold, physical effort, and exercise-induced fatigue. The sensitivity of respiratory rate to these conditions is superior compared to that of most of the other vital signs, and the abundance of suitable technological solutions measuring respiratory rate has important implications for healthcare, occupational settings, and sport. However, respiratory rate is still too often not routinely monitored in these fields of use. This review presents a multidisciplinary approach to respiratory monitoring, with the aim to improve the development and efficacy of respiratory monitoring services. We have identified thirteen monitoring goals where the use of the respiratory rate is invaluable, and for each of them we have described suitable sensors and techniques to monitor respiratory rate in specific measurement scenarios. We have also provided a physiological rationale corroborating the importance of respiratory rate monitoring and an original multidisciplinary framework for the development of respiratory monitoring services. This review is expected to advance the field of respiratory monitoring and favor synergies between different disciplines to accomplish this goal.

## 1. Introduction

A growing body of evidence suggests that respiratory rate, also known as respiratory frequency (*f*_R_), is a fundamental variable to be monitored in different fields. In healthcare, *f*_R_ is a vital sign which provides information on clinical deterioration, predicts cardiac arrest, and supports the diagnosis of severe pneumonia [1,2,3,4,5]. Furthermore, *f*_R_ responds to a variety of stressors, including emotional stress, cognitive load, cold, and hyperthermia [6,7,8,9]. During exercise, *f*_R_ is a good marker of physical effort and fatigue [10,11,12,13,14,15,16,17] and is associated with exercise tolerance in different populations [14,18]. Recent advances in the understanding of the control of ventilation corroborate the importance of monitoring *f*_R_ and explain why *f*_R_ but not tidal volume (V_T_) (the other component of minute ventilation) responds to a variety of non-metabolic stressors [7,11,12,17,19,20,21,22]. Likewise, technological development in the field of sensors and techniques for measuring *f*_R_ is growing exponentially, and a series of measurement solutions are currently available [23,24,25,26]. The ever-increasing interest in technological solutions for respiratory monitoring is manifested by the number of recent reviews published on this topic [23,24,25,26,27,28]. These reviews describe the advanced state of the art of the development of measurement systems for monitoring *f*_R_ and other ventilatory variables [23,24,25,26,27,28]. Nevertheless, one of the main challenges commonly highlighted is the limited use of respiratory systems in everyday-life monitoring. This issue is especially evident from the findings of a recent systematic review by Vanegas et al. [26]. Indeed, advances in respiratory physiology, applied sciences, and technology are not yet accompanied by a large diffusion of effective respiratory monitoring services in different fields. For instance, *f*_R_ monitoring is not performed routinely in healthcare or in the field of sport and exercise [14,24,26]. A major factor determining this limitation is the inadequate establishment of synergies between the different disciplines related to respiratory monitoring.

This review proposes the adoption of a multidisciplinary approach to respiratory monitoring as a solution to improve the development and efficacy of *f*_R_ monitoring services. We present a solid physiological rationale explaining why *f*_R_ is particularly sensitive to different non-metabolic stressors, thus corroborating the importance of *f*_R_ monitoring for different applications. Furthermore, we show how the understanding of the *f*_R_ response to different stressors facilitates the identification of suitable sensors and techniques for *f*_R_ monitoring in different measurement scenarios. Related implications for the development of measurement systems, algorithms, validation procedures, and respiratory monitoring services are discussed in this review. Briefly, Section 2 describes this approach in detail for different fields of use and applications, while Section 3 builds on such a multidisciplinary approach to propose an original framework for the development of respiratory monitoring services. Current challenges and directions for future research in the field of *f*_R_ monitoring are discussed in Section 4 and Section 5.

## 2. Goals and Measurement Scenarios Requiring Respiratory Rate Monitoring

**Figure 1:**
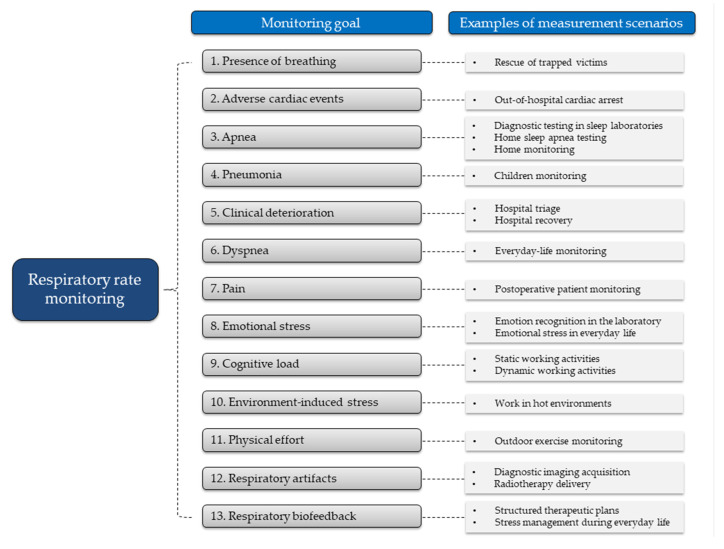
Schematic representation of the monitoring goals described in this review and related examples of specific measurement scenarios.

This section presents a series of monitoring goals where the measurement of *f*_R_ is invaluable, but with no attempt to cover all the potentially relevant applications. These goals are organized in different subsections, each of which is composed of two parts: (1) Current evidence; and (2) Measurement and Computing. The “Current evidence” sections present the importance of *f*_R_ monitoring for the specific goal identified, while the “Measurement and computing” sections describe suitable sensors and techniques to monitor *f*_R_ in specific measurement scenarios, which are taken as examples (see Figure 1 for a schematic representation). With this structure, we show how the choice of the *f*_R_ measurement technique depends on specific monitoring goals and measurement scenarios and is facilitated by the understanding of how *f*_R_ responds to different stressors. Accordingly, we provide the reader with specific examples on how to use available technologies for different applications and fields of use. When relevant, we also comment on the need to complement *f*_R_ monitoring with the measure of other ventilatory variables (e.g., V_T_), and on the physiological rationale underlying this need.

### 2.1. Presence of Breathing

#### 2.1.1. Current Evidence

Breathing is a vital physiological function of the human body. It guarantees gas exchange, acid–base balance regulation, and other homeostatic functions even under stressful conditions. As such, *f*_R_ is one of the most fundamental vital signs [1,29]. Normal *f*_R_ values (eupnea) range from 12 breaths/min to 20 breaths/min in adults [30], while the normal values for children vary according to age [31]. Different stressors acting on the human body determine variations in *f*_R_ outside the eupnea range, and this topic is covered in detail in the following subsections of Section 2. Differently, here we discuss the importance of detecting the presence of breathing per se, which has relevant implications for different fields of use. For instance, it is valuable for survivor identification in civil and military rescue scenarios [32] and for children below one year of age that are at risk of sudden infant death syndrome [33,34]. Furthermore, the assessment of breathing is fundamental in cardiorespiratory resuscitation. This evaluation is usually performed by manual counting, although even trained medical students and healthcare professionals may find this task challenging [35,36]. Hence, the objective measurement of *f*_R_ in cardiopulmonary resuscitation procedures might help in emergency management. While the accurate and objective monitoring of *f*_R_ would also prove of great value for a variety of other applications, *f*_R_ is often the least recorded vital sign [14,29,37,38].

#### 2.1.2. Measurement and Computing

The first requirement for any effective respiratory monitoring service is the need to obtain a good respiratory signal (respiratory waveform). This is particularly relevant when the aim is to detect the presence of breathing per se, as portions of low-quality signal may impair the possibility to unambiguously distinguish whether the user is breathing or not. However, this goal is complicated by the fact that the quality of the respiratory signal is influenced by numerous factors, including the type of sensors, the front-end/back-end electronics, the sensor(s) placement, undesired human movements, and environmental factors. A possible solution to address this issue is the assessment of the quality of the respiratory waveform before *f*_R_ values are obtained, as even a suitable and validated sensor may provide a low-quality signal under specific circumstances (e.g., misplacement of the sensors). While this approach is not yet common in respiratory monitoring, a quantitative assessment of the signal-to-noise ratio has been proposed by some researchers, with promising results. Given the indirect nature of *f*_R_ measurement from the electrocardiographic (ECG) and photoplethysmographic (PPG) signals, it is not surprising that such an approach of respiratory signal quality assessment has been used to a great extent when *f*_R_ is extracted from these signals. For instance, signal quality indices (SQI) are well-established indicators used to identify the presence of artifacts in the ECG and PPG signals and to improve the robustness of *f*_R_ estimation algorithms [39]. It has further been suggested that ad-hoc respiratory quality index algorithms based on Fast-Fourier Transform, Autoregression, Autocorrelation, and the Hjorth Parameter Complexity perform better than classical SQI in identifying poor-quality respiratory waveforms extracted from raw PPG and ECG signals and in estimating *f*_R_ [40]. In fact, the assessment of respiratory waveform signal quality can be applied to signals collected with a variety of respiratory sensors and is particularly useful for signal selection when different sensors are used simultaneously. An example is the evaluation of the quality of signals collected with different strain sensors attached to the chest and abdomen, which is particularly relevant when the respiratory signal is affected by motion artifacts during physical activity [41,42]. A similar approach was used by Siqueira et al. [43], who simultaneously recorded the respiratory waveform with multiple tri-axial accelerometers positioned on the chest and the abdomen. They found that a method based on independent component analysis was suitable to extract the respiratory waveform blindly, and that the quality of the respiratory signal was influenced by the sensor location [43]. The use of a SQI was also proposed for the quantification of the signal-to-noise ratio of respiratory signals recorded with a thermal camera [44]. The authors developed a SQI ranging from 0 to 1, which is based on four features that take both high-frequency and low-frequency noise into account [44].

The rescue of trapped victims is a typical example of a measurement scenario where the detection of the presence of breathing is of great value [32]. Contactless techniques can be used for victim identification, as the ultra-wideband (UWB) through-wall radar provides an estimation of *f*_R_, while calculating at the same time the distance between the radar and the human subject [45]. This feature of UWB radars is essential for survivor identification and location. However, a low signal-to-noise ratio can be found in complex environments and may result in significant errors in the estimation of *f*_R_ and distance. This problem can be counteracted with the development of robust algorithms as proposed by Shikhsarmast et al. [45], who implemented a random-noise denoising and clutter elimination algorithm using wavelet transform. Other approaches are based on complex signal demodulation techniques and frequency accumulation methods to suppress mixed products of the heartbeat and respiration signals and spurious respiration signal harmonics [46,47]. When the presence of breathing needs to be assessed, it is preferable to measure *f*_R_ on a breath-by-breath basis (see Table 1 for a summary).

### 2.2. Adverse Cardiac Events

#### 2.2.1. Current Evidence

Substantial evidence suggests that an elevated resting *f*_R_ is associated with cardiac arrest [1,2,48,49,50,51]. Indeed, *f*_R_ was found to be the most accurate vital sign to predict this adverse cardiac event [2,48,50,51], and this is why *f*_R_ has the highest weight in the cardiac arrest prediction model developed by Churpek et al. [2]. In this model, progressively higher scores are attributed to *f*_R_ values > 20 breaths/min, with the highest score assigned to values > 29 breaths/min [2]. Likewise, Fieselmann et al. [48] found that an *f*_R_ > 27 breaths/min was a better predictor of cardiopulmonary arrest compared to the heart rate and blood pressure in internal medicine inpatients, and other *f*_R_ thresholds were also predictive of cardiopulmonary arrest. The rise in resting *f*_R_ is observed hours before the occurrence of cardiac arrest [48,50,51], thus suggesting that *f*_R_ monitoring may help in the early detection and management of adverse cardiac events [1]. The prognostic power of *f*_R_ was also documented in patients with acute myocardial infarction, where *f*_R_ was found to be an independent predictor of the post-treatment outcome, with a doubling of mortality for every four-breath increment in *f*_R_ [52]. Furthermore, a study involving more than 900 patients with acute myocardial infarction found that nocturnal *f*_R_ (cut-off value > 18.6 breaths/min) was a good predictor of non-sudden cardiac death [53,54]. Likewise, a nocturnal *f*_R_ ≥ 16 breaths/min was found to be an independent predictor of long-term cardiovascular mortality in older adults [55]. The importance of these findings is not confined to healthcare settings but extends to in-home monitoring of patients at risk. Indeed, out-of-hospital cardiac arrest is a leading cause of cardiac death worldwide [56], and respiratory monitoring may aid the prediction or early management of such an event [57]. However, *f*_R_ is still poorly recorded in healthcare [29,38,58,59,60], despite substantial evidence of its clinical relevance. This contrasts with the ever-growing increase in technological development observed in the field of respiratory monitoring in the last years [23,24,25,26]. Therefore, we urge the improvement of respiratory monitoring services to help reduce the incidence of cardiac arrest and to lower the associated morbidity and mortality.

#### 2.2.2. Measurement and Computing

Prevention of out-of-hospital cardiac arrest is a vital monitoring goal for patients at risk. These patients may require continuous monitoring during everyday life and would benefit from vital sign measurement through wearable devices. Here, we present some techniques suitable to monitor *f*_R_ in a real-life scenario. Several technological solutions are currently available for the continuous monitoring of the ECG signal, including standard Holter devices, and sensors integrated into patches or garments [61]. When cardiopathic patients wear a device measuring ECG, it is tempting to extract *f*_R_ from this signal or to use ECG electrodes to measure *f*_R_ via impedance plethysmography. These two solutions have been commonly employed for respiratory monitoring, leveraging on the fact that no extra device is needed. The morphology of ECG is affected by breathing, which determines the amplitude, frequency, and baseline modulations of this signal [62]. The estimation of *f*_R_ from the ECG has proven to be successful in specific measurement scenarios, especially during nocturnal recording [53,54]. *f*_R_ estimated from the ECG of a Holter device was found to be a good predictor of non-sudden cardiac death, and this association was not substantially influenced by the number of ECG leads [53,54]. The same study showed good agreement between *f*_R_ derived from ECG and that measured with a piezoelectric sensor, but only when calculating the local maxima of different ECG-derived respiratory time series and not when using spectral analysis [54]. This suggests that the choice of the algorithm to process the ECG signal is critical. The nocturnal measurement of *f*_R_ from ECG was found to be suitable also in patients with sleep apnea [63]. Sleep monitoring for cardiopathic patients may also benefit from the recording of breathing sounds to assess the presence of agonal breathing, which is a frequent but under-appreciated diagnostic sign of cardiac arrest [57]. Machine learning algorithms have been developed to classify agonal breathing instances in real-time within a bedroom environment, with simulations showing a sensitivity of 97.24% and a specificity of 99.51% [57].

The estimation of *f*_R_ from ECG may present some problems during everyday-life activities. Indeed, the error in *f*_R_ estimation was found to be higher during a driving task compared to sleep, and increased for *f*_R_ values outside of the 0.1 Hz–0.4 Hz range [63]. An alternative approach is impedance plethysmography, where the ECG electrodes are used to detect respiratory-induced changes in thoracic impedance [24]. However, impedance plethysmography usually underperforms compared to techniques measuring respiration-related chest wall movements with strain sensors. This has been shown in different conditions, including exercise, ambulatory monitoring, and drug-induced respiratory depression [24,64,65]. Strain sensors (e.g., resistive, capacitive, and inductive sensors) may be suitable solutions to register the respiration-induced movements of the thorax or the abdomen and measure *f*_R_ continuously [24]. These techniques can provide real-time streaming of data for remote processing and visualization thanks to small electronics and connectivity capabilities [66].

Breath-by-breath *f*_R_ monitoring may not be strictly required for cardiopathic patients performing activities of daily life, and average *f*_R_ values over 60 s are sufficient in most cases. Conversely, the detection of agonal breathing requires the processing of the raw respiratory signal with machine learning algorithms [57].

### 2.3. Apnea

#### 2.3.1. Current Evidence

Sleep apnea is a serious breathing disorder associated with major neurocognitive and cardiovascular sequelae [67]. A causal relationship has been found between sleep apnea and the incidence and morbidity of hypertension, coronary heart disease, arrhythmia, stroke, and heart failure [68]. Furthermore, sleep apnea is associated with poor sleep quality, daytime fatigue, sleepiness, neuropsychiatric disorders (e.g., cognitive impairment and depression), and impairments in the quality of life [69,70]. Obstructive sleep apnea (OSA) is the most common form of apnea. It affects almost 1 billion people worldwide and its prevalence exceeds 50% in some countries [67]. Obesity is the major risk factor for OSA, but 20% to 40% of OSA patients are not obese [68]. Apnea events are differentiated from hypopnea events but both types concur to the computation of the Apnea-Hypopnea Index (AHI), which describes the severity of the disease [71]. An apnea event occurs when the airflow is absent or nearly absent (drop by ≥90% of pre-event baseline respiration) for at least 10 s, while hypopnea consists in a respiratory drop by at least 30% of pre-event baseline respiration for at least 10 s [71]. Hence, hypopnea detection requires the measurement or estimation of airflow (both *f*_R_ and tidal volume) [71]. The concomitant use of different sensors is needed for the differential diagnosis of OSA, central sleep apnea (CSA), or mixed sleep apnea, and different guidelines have been provided for children and adults [71]. However, most cases of obstructive sleep apnea remain undiagnosed and untreated, even in developed countries [67]. This is partially due to the laborious procedures required for the diagnostic testing of sleep apnea, which is usually performed overnight in sleep laboratories, involves high costs, and is uncomfortable for patients [72]. Hence, there is a growing interest in the development of cost-effective, noninvasive, and user-friendly solutions for the preliminary identification of sleep disorders or the home-monitoring of patients with sleep apnea [72,73]. Indeed, the timely diagnosis of sleep apnea and recognition of exacerbations can decrease morbidity, mortality, and the economic burden for healthcare systems. *f*_R_ monitoring plays an important role in achieving these goals.

#### 2.3.2. Measurement and Computing

The choice of measurement techniques for sleep apnea detection depends on specific monitoring goals and scenarios. Here, we describe some of the techniques used for: (1) diagnostic testing in sleep laboratories; (2) home sleep apnea testing; and (3) home monitoring. Diagnostic testing in patients suspected of having sleep apnea (polysomnography) is usually conducted overnight in sleep laboratories. The differentiation between OSA and CSA requires the simultaneous use of different sensors because the recording of chest and abdomen movements is required along with apnea identification. When these movements are present (i.e., the so-called “respiratory effort” is observed), the patient is diagnosed with OSA; otherwise, with CSA. Specific guidelines describe the measurement techniques needed as diagnostic tools for sleep apnea identification [71]. Apnea and hypopnea events are identified with the concomitant use of nasal pressure sensors and oronasal temperature sensors. Nasal pressure sensors provide a signal proportional to the square wave of the airflow and are sensitive to even subtle changes in airflow [71], although their sensitivity is higher at high flow rates compared to low flow rates. However, they may fail to detect or estimate oral airflow. This limitation is overcome with the simultaneous use of oronasal temperature sensors. These sensors (i.e., thermistor, thermocouples, pyroelectric and fiber optic sensors) show low obtrusiveness (a few millimeters in diameter), good response time (from some ms up to some seconds), and a high sensitivity to airflow in the temperature range of interest for respiratory monitoring [24]. On the other hand, the signal from temperature sensors is not proportional to the airflow, which determines an overestimation of low flow rates and an underdetection of hypopnea events [71,74]. While not considered by current guidelines, humidity sensors may provide a valid alternative to temperature sensors. Indeed, miniaturized relative humidity sensors (typically embedding nanocrystals and nanoparticles) exploit the water vapor differences between inhaled and exhaled air and are characterized by excellent response time (order of 40 ms) [24]. Besides, unobtrusive solutions based on hot-wire anemometers for direct oral/nasal airflow detection are promising and deserve consideration [75]. While apnea is usually detected with nasal pressure sensors and oronasal temperature sensors, the use of respiratory inductive plethysmography (RIP) (consisting of two belts positioned at the thorax and abdomen levels) or polyvinylidene fluoride sensors is recommended for “respiratory effort” detection [71]. However, other technologies based on conductive sensors (i.e., piezoresistive, piezoconductive, and capacitive sensors) are suitable for “respiratory effort” detection and should be considered in future guidelines. These sensors have been extensively reviewed by Massaroni et al. [24] and can be integrated into garments, belts, straps, and patches. One of the open challenges in the diagnostic testing of sleep apnea is the identification of hypopnea events, as the use of different criteria and sensors may result in marked differences in AHI values [76,77], with important implications for disease identification, severity grading, and clinical decision making.

A hot topic in sleep apnea research is the development of home sleep apnea testing procedures for the out-of-lab diagnosis, which requires the identification and use of less obtrusive solutions. Among the proposed technologies, tracheal sound measurement is a sensitive, reliable, and noninvasive technique [78,79,80]. When a microphone is placed at the suprasternal notch, tracheal sounds effectively detect sleep apnea events, even those missed by nasal pressure sensors due to mouth breathing or nose obstruction [79]. Hence, tracheal sound sensors meet the oronasal flow evaluation criteria for apnea detection required by the American Academy of Sleep Medicine, and can thus be used as alternatives to temperature sensors [79,80]. Furthermore, these sensors can provide additional useful information on snoring sounds and sleep/wake status discrimination [78,81].

Acoustic sensors can also be used in home settings when the aim is not to perform a diagnostic test for sleep apnea identification but to monitor the patient on a routine basis. To this end, sleep apnea can be detected with a mobile phone built-in microphone [81,82]. Other available techniques for apnea monitoring include the use of camera sensors for the recording of surveillance videos that can be post-processed to retrieve apnea episodes [83,84]. Besides, techniques based on instrumented items (e.g., sleep mats) have also been designed and tested, but further research is needed to improve their sensitivity to sleep apnea detection [85]. In patients with cardiac implants, Defaye et al. [86] provided a valid solution for night-to-night apnea monitoring using an implantable transthoracic impedance sensor. They observed a sensitivity of 100% and a specificity of 80% for sleep apnea and hypopnea detection, with important implications for the clinical management of this patient population [86].

Apnea detection requires the acquisition and storage of raw respiratory data because manual scoring is often performed [87]. On the other hand, several computing techniques have been used for the automatic detection of apnea, hypopnea, and related scores, including amplitude and adaptive thresholding, linear and kernel methods, tree based models, artificial neural networks, deep learning, and fuzzy logic systems and networks [87].

### 2.4. Pneumonia

#### 2.4.1. Current Evidence

Pneumonia is a leading cause of post-neonatal death in children under-five years [3,88]. The World Health Organization guidelines suggest that *f*_R_ should be integral to the pneumonia diagnostic pathway [3,88], especially in low- and middle-income countries, where timely pneumonia diagnosis is a much greater challenge because of limited resources [88]. This issue is of great relevance, considering that childhood pneumonia deaths could be prevented with simple interventions and appropriate treatments [89]. *f*_R_ cut-off values for severe pneumonia correspond to ≥60 breaths/min, ≥50 breaths/min, and ≥40 breaths/min for children who are <2 months of age, between 2 months and 11 months, and between 12 and 59 months of age, respectively [3]. Pneumonia is a serious infectious disease for other populations as well, including older adults [90,91] and patients with chronic obstructive pulmonary disease [92]. Furthermore, pneumonia outbreaks, as the pandemic caused by the SARS-CoV-2 virus (COVID-19 disease), constitute major medical, social, and economic challenges worldwide [93]. *f*_R_ monitoring may prove to be of great value in these circumstances, given the clinical relevance of *f*_R_ in the diagnosis, prognosis, and clinical management of COVID-19 [5]. Given the fact that *f*_R_ is altered substantially by pneumonia, *f*_R_ is among the variables used to define criteria for the diagnosis of severe pneumonia (≥30 breaths/min) and for the achievement of clinical stability (≤24 breaths/min) [94,95]. A large body of evidence suggests that *f*_R_ is an important prognostic marker and a predictor of mortality in patients with pneumonia [95,96,97,98], but not all the studies entirely support this notion [90,99]. Different findings between studies may be partially due to the fact that *f*_R_ is too often not accurately measured in the context of pneumonia [5,88,89,100]. Given the clinical relevance of *f*_R_ for the management of this disease, it is essential to use accurate systems for *f*_R_ measurement.

#### 2.4.2. Measurement and Computing

The COVID-19 pandemic has rapidly increased awareness of the importance of effective respiratory monitoring [5,101], which is an unprecedented opportunity to solve long-standing issues related to *f*_R_ monitoring in the context of pneumonia. Here, we focus on the measurement techniques suitable for pneumonia monitoring in children, a condition presenting some peculiar challenges, including high resting *f*_R_ values (especially in newborn babies) and the possible presence of artifacts in the respiratory signal due to movement and crying. A particularly relevant measurement scenario is that of pre-hospital settings in low-income countries, where the affordability of measurement systems and their simplicity of use are additional factors to take into account [88]. Methodological inconsistencies across studies have so far resulted in difficulties in the identification of suitable techniques to measure *f*_R_ in such a scenario [89,100]. Despite the important limitations of manual counting [88], this is still a commonly used method to measure *f*_R_ and is even selected as a reference method for validation studies [89]. Indeed, the choice of the reference system is a critical problem, as highlighted by a recent systematic review on the technological solutions available to measure *f*_R_ for pneumonia identification in children [89]. The authors reported great heterogeneity in the selection of reference systems, which may impact on the quality of some of the reviewed studies and limit the possibility to compare the performances of techniques tested in different studies [89]. Nevertheless, some contactless solutions appear promising [89]. Some of these technologies measure *f*_R_ from the detection of respiration-induced body movements, including depth sensors, radiofrequency sensors, and RGB (red, green, blue) camera sensors [23,102,103,104]. When the respiratory waveform is obtained from video image recordings, magnification algorithms can be used to improve the signal-to-noise ratio, especially when small movements of the chest wall are observed [102]. Alternatively, solutions based on the use of pressure or strain sensors embedded in mattresses or other bed components can be used to obtain accurate *f*_R_ values [105]. All these techniques are relatively cheap and can prove useful in non-collaborative subjects like newborns and children, with no need to attach sensors on the patient’s body. Thermal cameras and laser vibrometry sensors are other interesting solutions for the contactless monitoring of newborns in clinical scenarios [44,106], but their cost is relatively high [23]. On the other hand, contact-based solutions such as nasal pressure sensors, oronasal thermistors, and impedance plethysmography are currently used as diagnostic tools for sleep apnea in children [107]. These are suitable techniques for continuous *f*_R_ monitoring but are not practical for routine vital sign monitoring of patients suspected with pneumonia, especially in low-income countries.

Breath-by-breath *f*_R_ monitoring is not strictly needed in this context, and current UNICEF guidelines on diagnostic aids for acute respiratory infection require accuracy of ±2 breath/min over a recording period of 60 s [89]. While a series of contact-based and contactless techniques fulfill this requirement [23,24], so far their development and use have been limited by inadequate consideration of the specific needs of children living in low-income countries [89].

### 2.5. Clinical Deterioration

#### 2.5.1. Current Evidence

Evidence suggests that *f*_R_ is an important marker of clinical deterioration for a variety of pathological conditions in both adults [4,108,109,110] and children [111]. Indeed, *f*_R_ is a fundamental variable included in the majority of prognostic scores developed for the prediction of different outcomes, including intensive care unit (ICU) admission and mortality [4,109,110]. As such, *f*_R_ contributes to the computation of the most accurate prognostic scores developed so far, such as the National Early Warning Score (NEWS) and the Modified Early Warning Score (MEWS) [4]. The NEWS assigns a score to *f*_R_ values outside of the 12–20 breaths/min range, with the highest score attributed to *f*_R_ values ≤8 and ≥25 breaths/min [112], while the highest score for MEWS is attributed to *f*_R_ values ≥30 breaths/min [113]. A modified version of NEWS (i.e., NEWS2) has shown a good predictive capacity for the identification of in-hospital early mortality (all-cause) even when vital signs were collected at pre-hospital level, with *f*_R_ showing lower values in survivors compared to non-survivors [114]. *f*_R_ is also among the signs used for sepsis identification [115,116,117]. Furthermore, a nocturnal *f*_R_ ≥ 16 breaths/min is an independent predictor of long-term all-cause mortality [55]. A further increase in the accuracy of early warning scores is expected with measures performed at different time points as opposed to single measures [116,118], thus requiring devices to collect vital signs on a periodic or even continuous basis. This is important for timely critical care assistance because *f*_R_ may increase several hours before the occurrence of an adverse event [4,118,119], and such *f*_R_ changes should be promptly identified. However, despite the clinical relevance of *f*_R_, this vital sign is often under-recorded [29,37,120,121] or not measured accurately [38,59,60,122,123,124]. This may impair the efficacy of early warning scores [118,120,121], which also suffer from other methodological issues [109,110]. Therefore, it is imperative to improve the accuracy and frequency of *f*_R_ monitoring throughout the healthcare chain (pre-hospital, hospital, and post-hospital).

#### 2.5.2. Measurement and Computing

Vital signs are commonly measured during hospital admission at triage. However, *f*_R_ is measured by manual counting or is still too often not recorded at all [37,59,60,121]. The important limitations of this current practice have been discussed elsewhere [29,58,59,60,122,123,124,125]. This section presents some of the suitable techniques to measure *f*_R_ at hospital admission, with special attention to those allowing for periodic or even continuous monitoring of the patients needing hospital recovery. The extraction of *f*_R_ from the PPG signal is a practical solution as this signal is obtained from the pulse oximeter, which is routinely used in clinical settings to measure peripheral arterial blood oxygen saturation and heart rate. The pulse oximeter is usually applied at the finger (but also other locations can be used), is non-invasive, easy to use, and is suitable for the continuous monitoring of patients requiring special care. *f*_R_ can be extracted from the PPG signal because breathing affects this signal by determining the phenomena of baseline wander, amplitude modulation, and frequency modulation [62]. However, the occurrence of these phenomena depends on different factors, including breathing patterns, finger perfusion, health conditions, and body position [62]. This makes *f*_R_ estimation challenging and explains why a great body of research in this area is focused on the identification of computing solutions to improve the estimation of *f*_R_. A plethora of algorithms have been developed for the extraction and fusion of respiratory signals, for *f*_R_ estimation, for the fusion of *f*_R_ values obtained from different signals, and for quality assessment [62,126]. Given the indirect nature of *f*_R_ estimation from PPG, signal quality assessment is an important process requiring the assessment of both PPG signal quality and respiratory quality indices [62]. Indeed, the accuracy of *f*_R_ measurement is not only dependent on the quality of the PPG signal but also on the extent of breathing modulation.

Despite extensive research in this area, the implementation of algorithms estimating *f*_R_ from PPG is still not common in commercial devices. One of the exceptions is the Nellcor^TM^ Respiratory Rate Software application (Medtronic, Dublin, Ireland), which showed a good performance when tested in hospitalized patients against the capnography reference method (Mean of difference, MOD ± Limits of agreement, LOAs, 0.07 ± 3.90 breaths/min) [127]. Conversely, lower performances were found in the challenging measurement scenario of patients undergoing sedation and analgesia for endoscopy procedures, with a substantial difference observed between the *f*_R_ estimated from PPG with the Nellcor^TM^ 2.0 monitoring system (Covidien, Mansfield, MA, USA) and that obtained from capnography (MOD ± LOAs, 2.25 ± 10.60 breaths/min) [128]. Cardiac arrhythmias may also affect the physiological mechanisms responsible for the respiratory modulation of the PPG signal, and thus the quality of *f*_R_ measurement [62]. Nonetheless, the implementation of algorithms extracting *f*_R_ into commercial devices opens important avenues for *f*_R_ monitoring in clinical settings.

The current limitations of *f*_R_ measurement from PPG suggest that other techniques may complement the use of PPG devices at hospital triage. Contactless techniques have some practical advantages over contact-based techniques in this scenario, where the vital signs of several patients need to be recorded over a short period of time. Contactless techniques avoid the problem of sanitizing the measurement device after each use and generally make the patient less aware of the measurement, which matters because measurement awareness affects *f*_R_ values at rest [60]. Different sensors registering respiration-induced body movements can be suitable for this purpose, including depth sensors, camera-based sensors and radiofrequency sensors [23]. Depth sensors (e.g., Time-of-Flight sensors) are commercially available (e.g., Microsoft Kinect v2, Microsoft Corp., Redmond, WA, USA), provide an accurate measure of *f*_R_ when the patient is seated [129], and are less influenced by environmental factors (e.g., ambient light) compared to other contactless techniques [23]. Camera-based sensors and radiofrequency sensors (radar sensors and WiFi sensors) also show relatively good performances when measuring *f*_R_ in resting patients [130,131,132], and can be used to monitor different patients simultaneously [23]. However, further research is needed to assess the suitability of contactless sensors for *f*_R_ monitoring in hospital settings.

For patients needing hospital recovery, contact-based solutions allowing for continuous monitoring during a hospital stay may prove suitable, and some commercial devices have been developed for this purpose. Subbe and Kinsella [133] have assessed the validity of a wearable commercial device (RespiraSense™, PMD Solutions, Cork, Ireland) in patients admitted to the hospital as medical emergencies. This device measures respiration-related movements through a piezoelectric array located at the lower thorax level. On-board accelerometers and algorithms allow for the detection and partial removal of artifacts such as cough, speech, and motion artifact [133]. RespiraSense™ showed good accuracy when *f*_R_ (recorded over 15 min periods) was compared to capnography derived *f*_R_ [133]. This system can be worn for some hours and may increase the robustness of *f*_R_ measurement by selecting suitable (e.g., without motion artifacts) and multiple portions of the registered signal [133].

Two other FDA-approved wearable devices have been tested for validity, feasibility, and usability in patients admitted to the hospital and transferred to the general ward [118,134]. The ViSi Mobile system (Sotera Wireless, San Diego, CA, USA) measures *f*_R_ with impedance sensors attached on the chest [118,134], while the HealthPatch (Vital Connect, Campbell, CA, USA) is a disposable adhesive patch with reusable sensors, and extracts *f*_R_ from the ECG signal and the accelerometer signal [118,134]. Both devices were successfully used for the continuous monitoring of patients over 2–3 days of hospitalization, but the accuracy of *f*_R_ measurement was only tested against manual counting performed by nurses [118,134]. The discrepancy found between the *f*_R_ values measured with the ViSi Mobile and the HealthPatch and those collected by nurses impacted the computation of the MEWS [118,134], thus requiring further validation of the devices against an objective reference system. Use in real clinical settings also highlighted problems with connectivity, data loss, and artifacts affecting the signal [118,134], which requires consideration of the improvement and development of respiratory systems for patient monitoring in hospitals. The advantage of these techniques is the possibility to monitor the patient continuously throughout the healthcare chain, which greatly outperforms the current approach of manual counting over 60 s or even shorter periods of time [60]. However, more research is needed to improve the accuracy and suitability of respiratory devices for the assessment of clinical deterioration.

### 2.6. Dyspnea

#### 2.6.1. Current Evidence

Among the factors accounting for *f*_R_ being a marker of clinical deterioration, the association between *f*_R_ and dyspnea deserves consideration. Dyspnea is a major symptom in patients with chronic obstructive pulmonary disease (COPD) and other cardiorespiratory diseases [135,136], in obese individuals [137], and in older adults [138]. Furthermore, it is a major determinant of exercise intolerance and sedentary behavior in these populations, with consequent impairments in function and quality of life [135,137,138,139]. While dyspnea is a sensation of breathlessness (i.e., a symptom), an increase in resting *f*_R_ is its major physiological sign [140]. An association between *f*_R_ and dyspnea is observed both at rest and during physical exercise. At hospital admission, the resting *f*_R_ of patients admitted with dyspnea contributes to predicting the occurrence of different clinical outcomes, i.e., the use of non-invasive ventilation, ICU admission, and mortality [121]. The sensitivity of resting *f*_R_ as a predictor of COPD exacerbations is corroborated by findings from several studies [136,141,142,143], and is of paramount importance for the early detection and treatment of these adverse events. During exercise, a close association between *f*_R_ and dyspnea is observed in patients with different respiratory diseases, as similar responses are observed in patients with COPD and in those with interstitial lung disease [18].

Importantly, a neurophysiological link between dyspnea and *f*_R_ is evident because they are both regulated, at least to some extent, by the activity of areas of the brain relating to motor control, volition, cognition, and emotion processing [11,21,22,144,145,146]. On the other hand, dyspnea is a multidimensional sensation composed of three respiratory sensations with somewhat different underlying mechanisms and signs, i.e., respiratory effort, air anger, and chest tightness [145]. For instance, air anger is at least partially regulated by the magnitude of chemoreceptor afferent activity [147], and may thus be associated with a predominant increase in V_T_ [19,22,147]. An emblematic example is the air hunger associated with the deep and regular breathing observed in patients with metabolic acidosis, which is known as the “Kussmaul’s sign” [148]. Conversely, respiratory effort is at least partially regulated by the central motor drive to the locomotor and respiratory muscles (i.e., central command) [145,147], and may thus determine a predominant increase in *f*_R_ [13,22]. Given that patients present with various combinations of the afore-mentioned respiratory sensations [147], the monitoring of *f*_R_ and V_T_ may help shed some light on the pathophysiological mechanisms underlying dyspnea. As such, respiratory monitoring plays a fundamental role in the detection and management of dyspnea.

#### 2.6.2. Measurement and Computing

The assessment of the signs of dyspnea (e.g., an increase in *f*_R_) is particularly relevant during daily life activities (e.g., walking and stair climbing) where this symptom is exacerbated [149]. Here, we present some suitable measurement techniques for respiratory monitoring in this scenario. The need to monitor *f*_R_ during daily life requires the simultaneous identification of the activities performed by the patient [27]. Indeed, the severity of dyspnea is better described if the levels of *f*_R_ are interpreted along with the intensity and type of the physical tasks performed [149]. This information can be obtained from inertial measurement unit (IMU) sensors [27]. When located in specific parts of the trunk, IMU sensors may also be used to estimate both *f*_R_ and the respiratory amplitude [150,151,152]. By positioning accelerometers on the thorax and the abdomen, Fekr et al. [151] found that the use of a robust classification algorithm was suitable for the identification of eight different pathological breathing patterns, including the Kussmaul’s sign. However, the quality of the respiratory signal obtained from IMU sensors is largely affected by motion artifacts during physical activities [24]. On the other hand, IMU sensors can be used to improve the quality of the respiratory signal obtained with other sensors (e.g., strain sensors), through motion artifact identification and removal [24]. Therefore, it is preferable to complement the use of IMU sensors with other techniques for respiratory monitoring [24].

Strain sensors embedded into garments may prove particularly useful to measure *f*_R_ in patients with dyspnea, with a preference for those allowing for the estimation of V_T_ (or the respiratory amplitude as a surrogate) [24,153]. A smart garment designed for measuring physiological signs of dyspnea would benefit from the integration of strain sensors situated in specific locations of the trunk. Indeed, sensor redundancy improves the accuracy of *f*_R_ and V_T_ measurements [24,153,154], and may help detect other signs observed in patients with dyspnea such as the temporal thoracoabdominal asynchrony between the movements of the thoracic and abdominal compartments [155,156]. Thoracoabdominal asynchrony is often computed by means of the phase angle analysis, is higher during exercise compared to rest, and increases with exercise intensity [155]. Respiratory inductive plethysmography is a classical technique used to compute thoracoabdominal asynchrony with wearable sensors, and consists of two elastic cloth bands containing insulated wires encircling the rib cage and the abdomen [24]. Similar performances were found when comparing thoracoabdominal asynchrony measured with RIP and optoelectronic plethysmography (the reference system for measuring compartmental volumes [156]) in healthy individuals and patients with COPD and interstitial lung disease [155]. However, the agreement between the two techniques was higher at rest and during moderate exercise compared to heavy exercise, where a wide variability in the phase angle was observed [155].

Capacitive and resistive sensors also have metrological characteristics that are suitable for monitoring patients with dyspnea [24]. Naranjo-Hernández et al. [157] tested the feasibility of a remote respiratory service for monitoring the *f*_R_ of COPD patients during the recovery from home-based exercises. The measuring system was a smart vest embedding capacitive sensors, which showed superior performances (MOD ± LOAs, −0.14 ± 0.54 breaths/min) compared to those of some other measuring systems validated in the literature [157]. However, the authors did not assess the performances of the system during exercise, which is an important requirement for *f*_R_ monitoring in COPD patients and other patients presenting with dyspnea. Chu et al. [153] reported the good performance of small wearable piezo-resistive strain sensors situated at the level of the ribcage and the abdomen when *f*_R_ and V_T_ were compared with the same variables obtained with a spirometer. The wearable system was tested at rest and during ambulatory conditions, with interesting implications for the remote monitoring of patients with dyspnea [153]. However, the system was only tested on healthy individuals, and the respiratory signals were affected by motion artifacts (e.g., torsion of the trunk) during walking. Further research should focus on the development of wearable systems specifically designed for patients with dyspnea performing daily-life activities.

High-quality respiratory waveforms are needed to compute thoracoabdominal asynchrony and compartmental volumes. As such, it is preferable that respiratory systems measuring *f*_R_ in patients with dyspnea are validated on a breath-by-breath basis.

### 2.7. Pain

#### 2.7.1. Current Evidence

Pain is a leading cause of morbidity worldwide [158]. For instance, pain is a major healthcare issue in postoperative patients [159] and a common problem in patients requiring emergency medical service assistance [160,161]. It is well-established that pain influences breathing and generally determines an increase in minute ventilation [7,162]. This effect is mediated by an increase in *f*_R_, V_T,_ or both, depending on the nature of the painful stimulus [7,162]. The hormonal stress response which accompanies acute pain induces a predominant increase in V_T_ [163], while the psycho-behavioral changes induced by pain (e.g., discomfort, fear, and displeasure) affect *f*_R_ more. An example is the increase in *f*_R_ that occurs with the anticipation of pain before the advent of the nociceptive stimulus [164]. The stimulation of nociceptive afferents leads to a predominant increase in *f*_R_, which is documented by the elevated *f*_R_ observed in surgical patients under anesthesia [7].

In a cohort of over 50.000 patients with acute pain, Bendall et al. [165] found that an *f*_R_ > 25 breaths/min was the most important predictor of pain severity compared to other vital signs such as heart rate and blood pressure. Likewise, among different vital signs, *f*_R_ showed the strongest association with the severity of pain in over 18.000 patients requiring prehospital emergency medical service assistance due to pain [160]. It is also of note that *f*_R_ decreases with the administration of commonly used pain drugs (i.e., opioids), which makes *f*_R_ monitoring important to alert when the patient is at risk of respiratory depression [166], more so than arterial oxygen saturation measured by pulse oximetry [167]. This matters because opioid-related death is among the major causes of accidental mortality in adults [161], and brain damage may also occur [166]. Respiratory monitoring is also useful for the evaluation of pain in nonverbal critically ill patients or infants [162,168,169]. On the other hand, breathing may affect pain; several clinical and laboratory studies have reported a beneficial effect of slow deep breathing on pain [162]. Slow deep breathing may decrease pain perception through respiratory-induced cardiovascular/autonomic changes (e.g., respiratory sinus arrhythmia and variations in baroreflex activity), the modulation of cortical activity, and psycho-behavioral factors [162,170,171]. The effect of slow breathing on pain may improve with the use of respiratory biofeedback strategies [162,172]. Collectively, these findings suggest that respiratory monitoring is of great importance for pain detection and management.

#### 2.7.2. Measurement and Computing

A typical scenario where *f*_R_ can be used as a marker of pain is in postoperative patients. In this context, the main measuring challenge is the detection of respiratory depression, which may occur as a side effect of the administration of pain drugs (i.e., opioids), especially within 24 h of surgery [65,166]. Ermer et al. [65] conducted an interesting study specifically targeting the identification of suitable sensors capable of detecting *f*_R_ values below 10 breaths/min in sedated volunteers. Some methodological limitations of the study require caution in the interpretation of their findings, but useful information for further research have been provided [65]. The authors found that an abdominal accelerometer and a capnometer showed better performances compared to a nasal pressure transducer, an oronasal thermistor, a peritracheal microphone, transthoracic impedance sensors, and photoplethysmography [65]. The last two techniques listed showed the worst performances [65]. However, the sensors were validated against RIP, which may not be an ideal reference technique. This may partly explain the superior performances of the abdominal accelerometer, which was positioned in the same location of the abdominal RIP belt. Besides, a microphone may estimate *f*_R_ more effectively when located on the suprasternal notch [79] compared to a peritracheal location [65], and thermistors may underperform compared to other temperature sensors (e.g., pyroelectric sensors) [24]. Another possible limitation of the study is the use of the same algorithm to compare the performances of the different waveforms acquired with the various sensors [65]. In another study, the authors used the same data set to test the efficacy of a machine-learning algorithm in the identification of ataxic breathing severity, using breath-by-breath data of *f*_R_ and V_T_ collected with the RIP sensors and the nasal pressure sensor [173]. Given that alterations in ventilatory variability are commonly observed under the effect of opioids [173,174], the good performances of the support vector machine classifier tested by Elmer at al. [173] provide interesting perspectives on the identification of drug-induced irregular breathing. However, these findings [65,173] may not directly translate to everyday pain assessment as volunteers were asked not to talk or move and were monitored for relatively short periods of time, while postoperative patients require continuous monitoring [166,175]. Nonetheless, the study by Elmer et al. [65] highlights the importance of validating different sensors in a situation that resembles some of the characteristics of the measurement scenario of interest (i.e., opioid-induced respiratory depression).

While all the sensors tested by Ermer et al. [65] require direct contact with the patient’s body, less obtrusive techniques may also prove useful for the continuous monitoring of the *f*_R_ of patients suffering from pain. Isono et al. [176] tested an interesting solution for estimating *f*_R_ with four load cells placed under a medical bed. *f*_R_ was estimated by measuring the centroid shift in the cranio-caudal direction caused by the respiratory-related movements of the visceral organs. Accurate values of *f*_R_ were obtained in the range of 4 breaths/min to 40 breaths/min in different body positions, while *f*_R_ was underestimated above 40 breaths/min [176]. A similar solution with load cells under the bed proved valid for the estimation of apnea (100% sensitivity and 97% specificity) and hypopnea events [177], which makes this application suitable for respiratory depression detection. While non-respiratory movements may negatively affect the estimation of *f*_R_, the use of load cells facilitates the identification of movement artifacts.

Given the importance of detecting respiratory depression and irregular breathing induced by opioids, breath-by-breath monitoring of *f*_R_ and V_T_ is advised, although rarely performed, in the current clinical practice. Breath-by-breath monitoring and validation are also important requirements when measurement systems are used to alleviate pain through respiratory biofeedback. On the other hand, average *f*_R_ values over 60 s may provide sufficient information for the assessment of the pain-induced increase in *f*_R_. Along this line, the American Society of Pain Management Nursing Guidelines require that “respirations should be counted for a full minute and qualified according to rhythm and depth of chest excursion while the patient is in a restful/sleep state in a quiet unstimulated environment” [178].

### 2.8. Emotional Stress

#### 2.8.1. Current Evidence

It is well established that emotions affect ventilation, with a preferential influence exerted on *f*_R_ rather than V_T_ [8]. This is not surprising considering that *f*_R_ has been defined as the behavioral component of minute ventilation [19,20,22]. *f*_R_ increases with experimentally-induced anticipatory anxiety, unlike V_T_, oxygen uptake or carbon dioxide output [179]. This increase in *f*_R_ is positively related to individual trait anxiety scores [179]. Besides, *f*_R_ is sensitive to changes in affective valence and arousal [180]. This makes *f*_R_ a good candidate to identify emotional states in a variety of conditions and populations. For instance, *f*_R_ increases during panic attacks [7,181] and may discriminate between different pathological conditions; it is higher in patients with panic disorder compared to those with social phobia [182]. The fact that *f*_R_ is a good marker of emotional stress can be attributed to the fact that *f*_R_ is partially regulated by the activity of areas of the brain involved in emotional processing [8,183]. Indeed, direct stimulation of the amygdala produces a rapid increase in *f*_R_ [8]. On the other hand, the pattern of breathing influences emotions since voluntary breathing techniques (e.g., slow deep breathing) may attenuate negative emotional states [184]. Hence, the understanding of the interrelationship between breathing and emotions is fundamental to provide insight on how to treat anxiety, stress, depression, and emotional disorders [184].

#### 2.8.2. Measurement and Computing

When respiratory monitoring is purported to detect emotional stress, unobtrusiveness is an important requirement for the choice of the technique, as measurement awareness and obtrusive technologies may affect the individual emotional state and ventilatory responses [24,60]. Here, we present two measurement scenarios: (1) emotion recognition in the laboratory; (2) emotional stress detection in everyday life. In research laboratories, *f*_R_ is among the signs that may help recognize and classify emotions, along with heart rate, heart rate variability, galvanic skin response, body temperature, body posture, and facial expressions [180,185,186]. Contactless techniques are suitable for monitoring *f*_R_ in this scenario, and the use of techniques that can simultaneously record other relevant signals is particularly valuable. For instance, a thermal camera can be used to retrieve *f*_R_ and detect facial expressions at the same time from thermal video frames [187,188]. With this technique, *f*_R_ estimation is performed by analyzing respiration-induced changes in pixel intensity in a specific region of interest (at the level of the nose or mouth) [187]. However, the post-processing of video images is generally time consuming when compared to the majority of contact-based techniques, and infrared video images are usually analyzed after data collection. Other contactless sensors that can simultaneously register *f*_R_, face expressions and cardiovascular variables are RGB camera sensors and depth sensors [23,189,190]. When the area of the upper chest is filmed, RGB camera sensors can be used to retrieve respiration-induced body movements from the post-processing of video images [132]. Alternatively, if the face of the user is recorded with a camera, RGB camera sensors can be used to extract *f*_R_ from the modulation of the video PPG signal [189].

The understanding of the interrelationship between breathing and emotions depends on the accurate characterization of a number of respiratory features that can be extracted from the respiratory waveform [191,192]. Noto et al. [192] developed an open-source tool box (BreathMetrics) that automatically extracts a number of meaningful features embedded in human nasal airflow recordings. These include *f*_R_, V_T_, inspiratory and expiratory time, and inspiratory and expiratory pauses [192]. The use of the nasal flow measure was dictated by the close link between nasal flow and the activity of olfactory and limbic areas of the brain, but the authors are also trying to extend BreathMetrics functionality to respiratory waveforms obtained from sensors measuring the movements of the chest wall [192]. This would favor the recording of some important respiratory features in real-life scenarios. For instance, the possibility to record sigh events and ventilatory variability may further our understanding of the ventilatory response to emotional stressors [191]. Indeed, sighs and ventilatory variability are important elements in the regulation of breathing and emotions, with implications for the management of emotional stress and the prescription of therapeutic interventions in different diseases [191]. The respiratory waveform can also be analyzed with deep learning emotion recognition models, as good accuracy in the estimation of affective valence and arousal was found by Zhang et al. [180]. These findings open interesting perspectives for the real-life monitoring of emotional states.

Considering the aforementioned requirements, strain sensors recording the movements of the chest wall appear to be particularly suitable solutions to monitor emotion-related changes in *f*_R_ during everyday life, with a preference for resistive, capacitive, and inductive sensors [24]. The metrological characteristics of these sensors are detailed in a previous review by Massaroni et al. [24]. Strain sensors can be embedded into straps, bands, and t-shirts, and the electronics can provide real-time analysis and data streaming. Since the quality of the respiratory waveform affects the possibility of obtaining important respiratory features [192], it is preferable that the measurement systems used to detect the ventilatory response to emotional stress are validated on a breath-by-breath basis. This requirement is also needed for systems intended to provide ventilatory variability indices and respiratory biofeedback support for emotion management (see also the “2.13. Respiratory biofeedback” section).

### 2.9. Cognitive Load

#### 2.9.1. Current Evidence

It is well documented that *f*_R_, unlike V_T_, is sensitive to a variety of cognitive tasks and increases in proportion to the difficulty of the task [9]. It is, therefore, evident that *f*_R_ is the ventilatory variable that preferentially reflects cognitive load [9]. At rest, tasks like mental arithmetic, inhibition tasks, and working memory determine an increase in *f*_R_, with either no changes or a decrease in V_T_ [6,9,193,194,195]. Hence, *f*_R_ monitoring may help the detection of cognitive load in a variety of scenarios. This is particularly relevant for workers exposed to mentally demanding tasks and weighty responsibilities, including surgeons, soldiers, and pilots [9,195]. The variability of breath-by-breath *f*_R_ may provide additional insight into how *f*_R_ responds to cognitive load, but experimental evidence is scant and further studies are required to elucidate this issue [9]. The fact that *f*_R_ is sensitive to cognitive load is preserved during exercise; a cognitive task superimposed to physical exercise increases *f*_R_ compared to the sole physical task condition [196,197]. This has important implications for monitoring the extra load imposed by cognitive tasks during a variety of working and sporting activities that are characterized by different levels of psychophysical stress. The fact that *f*_R_ substantially responds to cognitive load suggests that *f*_R_ may at least partially be regulated by the activity of brain areas involved in cognitive processing. This input to ventilation has been defined as the “wakefulness drive to breathe”, i.e., an increase in central neural activity or arousal, similar to alertness or awareness [6]. While it has also been suggested that the increase in *f*_R_ may reflect the metabolic demand of the cognitive task [9], this interpretation is unlikely in light of the notion that metabolic inputs do not play a substantial role in the regulation of *f*_R_ [11,19,20,21,22]. Hence, *f*_R_ is a sensitive marker of the cognitive effort exerted in a task, with important implications for the health and performance of a variety of workers [198,199,200].

#### 2.9.2. Measurement and Computing

The quantification of cognitive load is of great relevance for numerous working activities. Here, we present measurement techniques that can be used to continuously monitor *f*_R_ during both static and dynamic working activities. Typical examples of workers reporting cognitive load under static activities are pilots, drivers, and computer workers [195,201,202]. As reported in a recent review of vital sign monitoring in automotive environments, a variety of techniques can be used for measuring *f*_R_ [203]. Indeed the car can be equipped with different sensors located in the seat, the backrest, the safety belt, the steering wheel, or the cockpit [203]. Interesting solutions include the use of strain/pressure sensors, camera-based sensors, and radar sensors [203]. Strain/pressure sensors have been used more often than the other solutions [203], and relatively good performances were reported in some studies [204]. Camera-based techniques are promising for obtaining accurate *f*_R_ values in this measurement scenario, but these solutions have received limited attention so far [203]. Several factors may explain the limited use of camera-based sensors in automotive environments [203], including privacy issues (especially when the face of the user is captured), the computational processing load of video images, and variable light conditions [203]. Another open challenge common to the afore-mentioned techniques is the susceptibility to motion artifacts (e.g., vibrations of the car). As such, Leonhardt et al. [203] suggest the simultaneous use of different respiratory sensors and the development of sensor fusion algorithms to provide a more robust measure of *f*_R_. Optical sensors, radiofrequency sensors, and strain/pressure sensors embedded in instrumented chairs are also suitable for monitoring computer workers [23,205,206,207,208]. Breath-by-breath *f*_R_ estimated from video recordings is generally more accurate compared to other contactless techniques, with errors below 4 breaths/min in the 10–40 breaths/min *f*_R_ range [206].

Cognitive load is also common in a variety of workers performing dynamic tasks, including soldiers [200] and healthcare professionals (e.g., nurses) [198]. Contact-based techniques are the best candidates to monitor *f*_R_ in these workers [24]. The sensors measuring chest wall movements appear more suitable than others, especially strain sensors. These sensors register changes in strain determined by respiration-related movements of the chest wall, and can be easily integrated into smart garments in the case of resistive, capacitive, and inductive sensors [24]. The accuracy of strain sensors is generally higher compared to that of contactless techniques (errors even lower than 1 breaths/min) [24]. Besides, the use of strain sensors is more suitable for breath-by-breath *f*_R_ monitoring compared to contactless techniques, as they require less computational resources compared to optical sensors, where a high quantity of information is processed to extract the respiratory waveform. Furthermore, strain sensors can be combined with other movement sensors (e.g., IMU) to reduce the influence of motion artifacts and improve the robustness of breath-by-breath *f*_R_ monitoring, even in real-time [24].

Breath-by-breath monitoring of *f*_R_ is required when attempting to gain insight into cognitive load by means of ventilatory variability analyses. Consequently, breath-by-breath validation is advised. Conversely, when monitoring cognitive load by means of *f*_R_ changes over time, average values over 60 s provide sufficient information.

### 2.10. Environment-Induced Stress

#### 2.10.1. Current Evidence

Evidence shows that *f*_R_ is very sensitive to different environmental stressors, including heat, cold, and hypoxia. Numerous studies suggest that *f*_R_ is the primary component of minute ventilation that responds to the heat stimulus [7,209]. A predominant increase in *f*_R_ with heat is observed both at rest and during exercise [7,209,210], where a good association between *f*_R_ and body temperature is generally found [210]. This association has important implications for the identification of workers at risk of heat strain [211,212], including those wearing protective garments (e.g., firefighters), those working in tropical climates, soldiers, agricultural workers, and individuals participating in major events organized in hot environments (e.g., sporting competitions). While the quantification of environmental factors (e.g., temperature and humidity) is useful to predict the risk of thermal strain, *f*_R_ monitoring is essential to understand the individual response to hot-environment exposure. Indeed, the attainment of critical levels of body temperature may derive from the combined effects of environmental-induced stress, equipment used and physical activity (a major source of body temperature increase) [212,213,214].

*f*_R_ is also sensitive to cold-induced stress, especially when sudden cold occurs. An emblematic and dangerous condition is the response to cold water shock, where *f*_R_ increases very rapidly and reaches values even higher than 60 breaths/min [7,215]. Conversely, a preferential increase in V_T_ is observed under prolonged cold as a result of the metabolic demands of shivering [7,19]. On the other hand, *f*_R_ reflects a cold-induced reduction in exercise capacity, as it increases with cold water immersion [216] and prolonged rain [217] compared to control conditions. These findings have implications for the monitoring of workers operating in cold conditions, including soldiers and maritime workers [218]. *f*_R_ is also sensitive to hypoxia both at rest and during exercise [7,219], with important implications for individuals working in low oxygen environments [220,221]. Therefore, *f*_R_ monitoring is fundamental for workers exposed to a variety of environment-induced stressors, both in terms of health safety and work productivity.

#### 2.10.2. Measurement and Computing

The need to face environment-induced heat strain is a typical requirement for individuals working in challenging environments. Here, we present the main measurement techniques suitable for *f*_R_ monitoring in hot environments. Some of the workers facing heat challenges wear masks as personal protective equipment. Examples are self-contained breathing apparatus used by firefighters or soldiers [222] and face masks used by healthcare professionals facing outbreak challenges (e.g., the 2013 Ebola virus West Africa outbreak) [214]. A variety of sensors can be integrated within a mask for *f*_R_ monitoring. These include airflow sensors (e.g., miniaturized pressure sensors and hot-wire anemometers), temperature sensors (e.g., thermistors, thermocouples, and pyroelectric sensors), humidity sensors, and acoustic sensors [24]. The performances of recently developed humidity sensors deserve special consideration in this context. He et al. [223] reported that graphene nanochannels confined poly-dopamine humidity sensors embedded in a mask show high sensitivity, ultrafast response (20 ms), and little humidity hysteresis. These performances were not substantially affected by high relative humidity (~75%), wind (up to 10 m/s) or physical activity [223]. Furthermore, the same sensors may even be capable of voiceprint recognition [223], thus making it possible to recognize when the respiratory signal is affected by speech without the need for additional acoustic sensors. This is an important feature for the continuous monitoring of *f*_R_ in real-life working scenarios.

For those individuals not wearing protective masks, *f*_R_ can be monitored with sensors embedded in belts or garments. Different commercial devices have been developed for vital sign monitoring in occupational settings. These include Zephyr^TM^ BioHarness^TM^ (Zephyr Technology Corporation, Annapolis, MD, USA) (i.e., a belt embedding capacitive sensors) [224], Equivital^TM^ EQ02 LifeMonitor (Hidalgo, Cambridge, UK) (i.e., a belt embedding inductive sensors) [225], LifeShirt^TM^ (Vivometrics, Ventura, CA, USA), and Hexoskin^®^ (Carre´ Technologies Inc., Montreal, Canada) (i.e., shirts embedding inductive sensors) [226,227]. These devices generally show good accuracy for *f*_R_ measurement even during exercise [14]. The performances of the Zephyr^TM^ BioHarness^TM^ were also tested during 40 min of submaximal exercise in a hot environment and found to be relatively good (MOD ± LOAs, 0.2 ± 8.3 breaths/min), but not as good as those observed during exercise in temperate conditions (MOD ± LOAs, −0.6 ± 5.0 breaths/min) [224]. This difference is possibly due to the fact that moisture affects the properties of the capacitive sensors [224]. Besides, the comfort of some of these devices could be improved, and sensors directly integrated into smart textiles are attractive alternative solutions.

Several factors should be considered when developing smart clothing for hot environments and extreme environments in general. Not only may different sensors change their properties with environmental factors (e.g., temperature and humidity), but conductive wires may also be affected, depending on the fabric of the smart textile [228]. When dealing with the choice of suitable sensors, the use of fiber optic sensors is encouraged, as their performance is not affected by changes in relative humidity [229]. However, despite recent advances in the field of respiratory monitoring with fiber optic sensors [28,229], further research and development are needed to use this technology during real-life working activities [28,229]. In an attempt to characterize the performances of smart textiles in challenging environments, Torreblanca González et al. [228] have developed a methodology for testing the effect of environmental factors on specific components of a smart textile. This methodology or similar approaches should be used to guarantee the correct functioning of smart garments designed for *f*_R_ monitoring in challenging environments.

In most of the cases, breath-by-breath *f*_R_ monitoring is not necessarily required for the detection of environment-induced stress, and data averaged over 60 s provide sufficient information. As such, most of the commercial devices used in occupational settings have been validated over 60-s long time windows [224,226,227]. More detailed information (e.g., 10-s average *f*_R_ values) may be required for specific needs, like for a proper description of the cold shock response [215].

### 2.11. Physical Effort and Fatigue during Sport and Exercise

#### 2.11.1. Current Evidence

As recently reviewed by Nicolò et al. [14], *f*_R_ is one of the most important variables to be monitored during sport and exercise. It is closely associated with perceived exertion during exercise protocols with different durations, formats (e.g., continuous and intermittent) and modalities (e.g., cycling and running) [10,12,13,15,16,230], at least during high-intensity exercise [11]. Furthermore, it is associated with exercise tolerance under a variety of experimental conditions, including hyperthermia, cold, hypoxia, muscle damage, muscle fatigue, dietary-induced glycogen depletion, respiratory muscle fatigue, and prior exercise [14,20,21]. Conversely, other physiological variables such as oxygen uptake, blood lactate, and heart rate may not be associated with perceived exertion and exercise tolerance in at least some of the aforementioned conditions [10,12,13,14,16,21]. Furthermore, unlike other physiological variables, *f*_R_ shows a rapid response at exercise onset and offset [12,14,15,231], with important implications for monitoring intermittent-based activities like soccer and other team sports [15]. As such, *f*_R_ provides invaluable insight into physical effort, and its time course reflects exercise-induced fatigue in different populations [10,12,13,14,15,16,18,21].

The fact that *f*_R_ is a valid marker of physical effort is corroborated by our current understanding of the control of ventilation [22]. During high-intensity exercise, the central motor drive relating to voluntary muscle contraction (i.e., central command) is a major regulator of *f*_R_ [11,12,13,17,21,22]. This is interesting considering that central command is also the primary regulator of perceived exertion [22,232,233], thus explaining the close association between these two variables [11,12,13,14,22]. In fact, *f*_R_ has several advantages over perceived exertion monitoring as it is an objective physiological variable that can be monitored continuously and in real-time, and provides detailed information on how physical effort is distributed over a given training session or more [14,15]. When maximal effort is exerted, *f*_R_ reaches peak values of about 50 breaths/min in the general population [234] and of about 60 breaths/min in athletes [12,13] ranging from 20-29 years old, but higher *f*_R_ peak values can also be observed [15,235]. The *f*_R_ peak shows a 5% decrease per subsequent decade and slightly lower values in females than males (the difference is 2 breaths/min on average), while it is not affected by stature [234]. However, inter-individual differences in *f*_R_ values [14,15,235] imply that *f*_R_ monitoring should be tailored on an individual basis for training optimization and performance assessment. This goal can be achieved with the routine use of accurate respiratory wearables specifically designed for exercise monitoring.

#### 2.11.2. Measurement and Computing

The importance of *f*_R_ monitoring in sport and exercise is not currently followed up by widespread use of respiratory devices in training and competition settings. This is partially due to the fact that *f*_R_ has only recently emerged as a fundamental variable to be monitored in the field of sport [14]. Indeed, it has even been defined as “the neglected physiological measure” during exercise [14]. However, there is also a paucity of wearable solutions specifically designed for exercise monitoring [14], which poses several measurement challenges. Indeed, sport-specific movements, changes in body posture, and physical contact with team members and opponents (e.g., in team sports) determine a variety of motion artifacts that may impair the quality of the respiratory signal [236]. Furthermore, exercise presents some thermoregulatory challenges (e.g., increases in body temperature and consequent sweating) that need to be considered in the choice of sensors, textiles, and components of the measurement system. Outdoor exercise monitoring is even more complex as environmental factors, including rain, snow, wind, humidity, temperature, and noise may constitute further obstacles for using some measurement techniques. This may be the case of the contactless methods [23], and of the contact-based methods based on air temperature, air humidity and acoustic sensing [24]. On the other hand, the abundance of technological solutions for measuring *f*_R_ makes exercise monitoring entirely feasible if the sports industry sector devotes efforts in this direction.

We present here some suitable techniques to monitor *f*_R_ in the challenging measurement scenario of outdoor exercise. With the aforementioned considerations in mind, the contact-based techniques measuring the movements of the chest wall appear to be good candidates [24]. As such, it is not surprising that most of the commercially available solutions tested during exercise use these techniques [14], with a preference for strain-sensitive conductive sensors. Among these, the most frequently used sensors are resistive, inductive, and capacitive sensors [14]. Different commercial devices have been validated during exercise, and good performances were generally reported [14,224,227]. However, in most of the cases, these devices were only validated in the laboratory, and less is known on the feasibility and suitability of their routine use in applied scenarios, like during outdoor training [237]. This is partially due to the fact that these devices were not specifically designed for sporting activities and that their wearability needs to be improved in some instances [14]. Besides, validation during exercise is rarely performed on a breath-by-breath basis, even though this is an essential requirement for real-time respiratory monitoring [238]. Furthermore, detailed information on *f*_R_ (i.e., average values over 3–5 s) describes the rapid *f*_R_ changes that occur during intermittent-based activities and provides insight on how effort is distributed during exercise [14,15]. Therefore, breath-by-breath *f*_R_ validation is strongly advised for sports respiratory wearables [238].

Another critical factor when designing sports wearables is the choice of the sensor position. Suitable body locations may partially change with exercise modality due to sport-specific postures and movements, with clear differences observed when comparing cycling, running, rowing, and swimming. Unfortunately, this problem has been overlooked, and only a few studies have attempted to address this issue so far [42,154]. While the abdominal rib cage appears to be a good body site to locate respiratory strain sensors during both running and cycling [42,154], sensors located on the abdomen showed good performances in cycling [154] but not in running [42]. Differently, sensors located in the upper thorax showed lower performances compared to those positioned on the abdominal rib cage both in cycling and running [42,154]. Besides, the posterior side of the trunk (both at the abdominal rib cage and abdomen levels) appears to be a suitable location that deserves consideration during cycling exercise [154]. However, these are only preliminary findings that need to be corroborated and expanded by future research.

Studies testing the performances of multi-sensor measuring systems are also valuable to shed some light on the influence of the number of sensors on *f*_R_ accuracy. Indeed, sensor redundancy is advised to improve the robustness of *f*_R_ measurement, and this is suggested by several studies performed both at rest and during exercise [41,42,154]. Sensor fusion with other sensors (e.g., inertial sensors) may also be beneficial for motion artifact identification and removal [42]. This requires the development of ad-hoc and adaptable algorithms resilient to breathing-unrelated movements [41,42], which is an important area of computing research for exercise monitoring. On the other hand, sensor redundancy may determine an increase in battery consumption and, therefore, a trade-off needs to be found depending on the specific application.

Some of the aforementioned challenges can be overcome with the advent of a new generation of sensors and electronic components. Stretchable and flexible sensors and electronics are particularly suitable for exercise monitoring as they ensure good adhesion with the body while exerting a minimal mechanical load on athletes [239]. Furthermore, the possibility to cover the stretchable system with moisture-resistant barrier layers and coatings help limit failure in functionality caused by sweat excretion and fluid exposure [239]. Strain stretchable sensors can be designed in various forms [240,241], which offers a myriad of solutions to satisfy specific measurement needs. These systems can be integrated into garments, patches, or can even be directly applied to the skin. For instance, Yang et al. [242] developed and tested an epidermal electronic system composed of metallic sensors connected by gold-on-polyethylene terephthalate serpentine ribbons, and promising results were observed for *f*_R_ measurement. The development of these wearable devices has been favored by advances in microelectronics and the use of intrinsically stretchable and flexible materials [239]. Other interesting solutions proposed for *f*_R_ measurement are textile-based sensors, conductive yarns, and highly-sensitive graphene strain sensors [240,243,244]. Facing the measurement challenges posed by sport and exercise has several advantages that go beyond this field of use and the sports industry sector. Indeed, accurate respiratory wearables suited for exercise monitoring can easily be scalable for everyday-life monitoring of patients, workers, and other users.

### 2.12. Respiratory Artifacts

#### 2.12.1. Current Evidence

A highly relevant field of research is the identification and compensation of respiratory artifacts to improve other biological measurements. The alternation of inspiration and expiration determines periodic movements of the organs situated in the abdominal and thoracic cavities, hence impairing the recording of biological signals coming from these body sites. Most of the imaging techniques used for medical diagnosis suffer from this problem, with specific challenges for different techniques. For instance, the time required to image the thorax is different for Positron Emission Tomography (PET) (6–9 min) and Computed Tomography (CT) (~15 s) [245], which increases the difficulty of combining images acquired with the two different techniques [245]. Hence, it is not uncommon to observe between-image spatial misalignments, which may result in the mislocalization of a tumor lesion or the inaccurate quantification of indicators used as criteria for malignancy [245]. Magnetic Resonance Imaging (MRI) quality is also impaired by respiratory artifacts [246]. As such, research in the area of respiratory artifact compensation is growing exponentially, and a variety of technological solutions are currently available for the improvement of imaging for medical diagnosis [247,248]. Likewise, the management of respiratory artifacts is essential for some therapeutic fields, and especially for radiotherapy [249]. Indeed, the side effects of radiotherapy are reduced if the patient’s breathing pattern is taken into account, and a consequent improvement in therapy effectiveness can also be observed. An example is the use of the deep inspiration breath-hold technique in breast cancer patients, which reduces the radiation dose to healthy organs at risk, including the heart [249]. The deep inspiration moves the heart away from the radiotherapy beam, and the breath-hold maneuver minimizes respiratory movements. This and other breathing techniques used for radiotherapy (e.g., respiratory gating) are performed with the help of respiratory biofeedback [250], thus making respiratory monitoring essential. Other biological signals are also affected by respiratory artifacts, including the ECG and the electroencephalographic signals, and different approaches have been proposed to address this issue [126,251,252,253]. Hence, respiratory monitoring is fundamental for improving the quality of a variety of biological signals and the management of different diseases.

#### 2.12.2. Measurement and Computing

The accurate and robust measurement of the respiratory waveform (not only of *f*_R_) is fundamental to counteract the challenges posed by respiratory artifacts. Here, we present some suitable techniques to record the respiratory waveform during imaging acquisition for diagnostic purposes, and during radiotherapy delivery. A variety of contact-based and contactless techniques can be used to improve diagnostic imaging, but the materials composing the measurement systems need to be compatible with magnetic fields. Another requirement is the connection between the measurement system and the controller of imaging device scans (e.g., CT, PET, and MRI). The simultaneous recording of diagnostic images and of the respiratory waveform allows for respiratory artifact removal through motion compensation algorithms [245]. In most of the cases, the respiratory waveform is obtained with sensors capable of measuring respiratory-induced phenomena. Some examples are pressure sensors, airflow sensors, temperature sensors, and the Real-Time Position Management Respiratory Gating System (Varian Medical Systems, Palo Alto, CA, USA) [245]. Other approaches are based on recording the respiratory waveform directly from CT or MRI scans, without the need for additional respiratory sensors. For instance, Shahzadi et al. [246] tested the efficacy of three different methods of respiratory motion detection and compensation using data directly acquired from MRI under free-breathing conditions. The methods were based on the Golden-Angle Radial Sparse Parallel MRI technique that combines parallel imaging and golden-angle radial sampling. These methods showed good performance and the possibility to sort the data into different respiratory phases, with their suitability depending upon the specific clinical application [246]. The presence of respiratory artifacts is particularly challenging in combined PET/CT imaging because of the different acquisition time of PET and CT and of possible misalignments between images. Given the abundance of methods for correcting motion in CT and PET images, the interested reader is referred to a previous review [245] for detailed information on this topic. With the technological advances of imaging in resolution and quality, an ever-increasing demand for respiratory artifact compensation solutions is expected in the next future [247].

Numerous techniques and algorithms used to improve diagnostic images’ quality are also suitable for respiratory artifact management during radiotherapy delivery. In this measurement scenario, it is even more important that the respiratory waveform is recorded in real time, and the use of respiratory biofeedback is common [250]. Different technologies based on direct contact with the body are available for this purpose. Among others, commercial devices based on airflow measurement are used in clinical practice to help patients perform apnea maneuvers during radiotherapy. An example is the Active Breathing Coordinator developed by Elekta (Elekta Oncology systems Ltd, Crawley, West Sussex, UK), an apparatus consisting of a turbine flow meter and a balloon valve capable of enforcing patient breath-holds at preselected respiratory volumes [254]. While methods based on airflow measurement are accurate, they are also obtrusive and sometimes not tolerated by patients.

In some radiotherapy procedures, the recording of the chest wall position is of additional value to address the problem of inaccurate radiation dose delivery caused by respiratory movements. Some of the technologies used to track respiration-induced chest wall movements during radiotherapy have been reviewed by Glide-Hurst and Chetty [255]. Among these, pressure-sensitive belts, infrared tracking systems, and camera-based sensors are commonly employed in clinical practice [255]. An example of a pressure-sensitive belt solution is the Anzai Respiratory Gating System (Anzai Medical Co. Ltd, Tokyo, Japan) [256]; the sensitive element is located at the right upper quadrant of a patient’s abdomen and the system includes two pressure sensors (with different sensitivities for patients with shallow vs. deep respiration amplitudes) [256]. The CyberKnife (Accuray, Sunnyvale, CA, USA) is an example of an infrared tracking system used to record the trajectories of hemispherical photo-reflective markers taped on the skin of the patient undergoing stereotactic radiosurgery [257]. Respiratory gating techniques have also been implemented with the use of an infrared respiratory camera tracking the movements of a reflective marker box placed on the patient’s abdominal surface [258]. Another alternative technique for respiratory motion compensation during radiotherapy is based on ultrasound motion tracking [259,260]. The ultrasound system used by Ting et al. [259,260] records respiratory movements at 30 Hz, with a total delay time of approximately 350 ms. Together with the use of respiratory motion algorithms, these performances may favor a reduction in the size of the planning target volume margin and an increase in the accuracy of the radiotherapy dose delivery [260].

The use of markers for tracking chest wall movements may suffer from the disadvantage of relative motion between the skin and the tracking markers [261]. This problem can be overcome with the use of video markerless approaches. Numerous optical systems that do not require markers are commercially available, including the AlignRT/GateCT (VisionRT Ltd, London, UK), the Sentinel (C-RAD AB, Uppsala, Sweden), and the Galaxy systems (LAP Laser, Luneburg, Germany) [261]. The AlignRT optical system was used by Schaerer et al. [261] to record the chest wall movements at three different phases of the breathing cycle (i.e., maximum inhale, maximum exhale, and an arbitrarily chosen intermediate position) and to develop an algorithm suitable for respiratory artifact removal. Depth sensors can also be used for respiratory motion tracking during radiotherapy. A good agreement was found between the Microsoft Kinect v2 and the more commonly used Anzai Respiratory Gating System and Varian’s RPM system [262]. The characteristics and ease of use of depth sensors [262] provide interesting avenues for improving radiotherapy management.

### 2.13. Respiratory Biofeedback

#### 2.13.1. Current Evidence

The voluntary modulation of breathing leads to a series of systemic effects inducing potential benefits in a range of disorders. For instance, slow breathing decreases blood pressure in patients with hypertension [263], reduces stress, anxiety, and pain [264,265,266,267,268,269,270], reduces the frequency and severity of migraine headaches [271], and improves various aspects of health-related quality of life in heart failure patients [272,273]. These effects are best achieved if the voluntary modulation of breathing is performed via respiratory biofeedback, which facilitates the maintenance of a given breathing pace or the execution of specific breathing exercises. *f*_R_ is a fundamental variable for any respiratory biofeedback strategy, but other ventilatory variables may also prove useful, such as V_T_, inspiratory time, and expiratory time [274,275]. The primary role of *f*_R_ in respiratory biofeedback is given by the marked effect of its change on different physiological systems, including the modulation of heart rate variability, which is particularly effective at *f*_R_ values around 6 breaths/min [276]. It has even been suggested that the effect of respiratory modulation on heart rate variability is maximized when *f*_R_ is set at the so-called individual resonant frequency, but more research is needed to further test this hypothesis [276]. Respiratory biofeedback is usually delivered by sound output and/or visual feedback, including attractive forms like music and biofeedback games [275,277,278]. It is a very useful technique to learn respiratory skills and exercises (e.g., diaphragmatic breathing) [271], especially for less compliant individuals like children and older adults. For instance, respiratory biofeedback is effectively used in tumor patients undergoing radiotherapy, where it favors therapy delivery while minimizing the therapy side effects [250]. Therefore, respiratory biofeedback has important implications in various settings, including clinical, rehabilitation, occupational, and leisure settings. Finally, research on respiratory biofeedback is very promising as it may help reconsider the role of respiratory devices, which may offer therapeutic solutions along with respiratory monitoring.

#### 2.13.2. Measurement and Computing

The essential requirement for any respiratory biofeedback system is the real-time display of the respiratory signal, either in the form of raw data (respiratory waveform) or in the form of ventilatory variables (e.g., *f*_R_, V_T_, inspiratory time, and expiratory time). In most cases, the patient is required to match a predefined respiratory pattern template by altering breathing voluntarily. Visual, auditory, or other forms of feedback are provided to help the patient accomplish this task. The choice of the respiratory measurement technique depends on the specific goal to achieve and on related measurement requirements. Here we provide an example of two measurement scenarios, where the patient performs (1) breathing exercises as part of structured therapeutic plans; or (2) biofeedback-guided exercises for everyday-life stress management.

Respiratory biofeedback is particularly relevant for patients performing breathing exercises as part of their therapeutic plans. This is the case for patients undergoing radiotherapy, who need to perform breathing familiarization sessions before real therapy sessions. Commercially available depth cameras (e.g., Microsoft Kinect) are good and relatively low-cost solutions to help patients practice with breathing exercises and procedures. When assessing the reproducibility of breathing maneuvers used for image-guided interventions, Heerink et al. [279] found that respiratory biofeedback delivered with the Microsoft Kinect v1 camera (Microsoft Corp., Redmond, WA, USA) was effective in reducing the respiratory motion variability observed when no biofeedback was used, with important implications for radiotherapy delivery. The patient undergoing radiotherapy is also advised to perform biofeedback-guided respiratory training outside of the clinical setting, although this practice is not performed regularly. In an attempt to partly overcome this limitation, Oh et al. [280] have developed a respiratory biofeedback system based on a micro-electro-mechanical-system magnetic sensor. This system showed good performances and relatively small errors in respiratory frequency and amplitude quantification [280]. However, the high magnetic fields generated by this system limit its applicability in some populations, including patients with pacemakers [280]. The use of other sensors registering respiration-induced chest wall movements (e.g., strain sensors) [24] can overcome this problem.

Other patients that would benefit from respiratory biofeedback therapies are those with hypertension [263,273,281,282,283] or panic disorders [284,285]. Substantial evidence shows a significant reduction in blood pressure with the administration of biofeedback-guided slow deep breathing exercises [273,281,282]. A number of these studies have used an FDA-approved commercially available biofeedback system called RESP*e*RATE^®^ (Intercure Inc, Fort Lee, NJ, USA) [273,281,282,283], which interactively reduces the patient’s *f*_R_ through auditory feedback with different tones for the inhalation and exhalation phases. This system is suitable for self-treatment at home and is made up of a sensor registering chest-wall movements embedded in an elastic belt, a computerized display, and headphones [282]. Differently, patients with panic disorders are usually treated with capnometry guided respiratory biofeedback because these patients hyperventilate and need to restore isocapnia [284,285]. Capnometry guided respiratory biofeedback provides values of end-tidal carbon dioxide and *f*_R_ to the user. Tolin et al. [285] have documented the feasibility of a remote monitoring service based on home-delivered capnometry-guided biofeedback therapy. The measuring system consisted of a CO_2_ sensor, a nasal cannula, and an app installed on a smart device that provided real-time audiovisual feedback of the variables of interest. Data from each session were streamed on a secure service after each treatment, thus allowing the clinician to remotely evaluate clinical progress and therapeutic adherence [285].

Respiratory biofeedback is also gaining interest in stress management during everyday life [275]. For instance, technological solutions may help face acute events of stress, anxiety, or panic. This need requires the use of unobtrusive technologies, with smart garments being particularly suitable as the user can access respiratory biofeedback as needed. Several sensors may be integrated into shirts or bands, including inductive, resistive, capacitive, and impedance sensors [24]. Among these, sensors with good response time and an output proportional to airflow should be preferred, as respiratory biofeedback delivery is more effective when respiratory amplitude (surrogate measure of V_T_) values are provided together with *f*_R_ values. Most of the solutions proposed so far have used sensors embedded in belts [278,286,287], but smart clothing may offer additional benefits in terms of wearability [288].

## 3. A Conceptual Framework for the Development of Respiratory Monitoring Services

**Figure 2:**
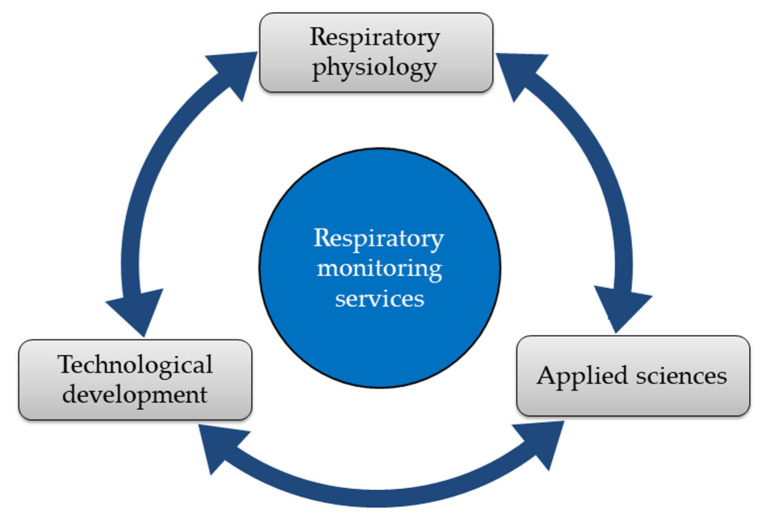
Schematic representation of the interactions between respiratory physiology, applied sciences, and technological development. The Figure shows how fruitful synergies between different disciplines are essential for the development of respiratory monitoring services.

The previous section has highlighted the importance of respiratory monitoring for different goals and measurement scenarios, thus pointing to the need to approach respiratory monitoring from a multidisciplinary perspective. Indeed, current advances in the fields of respiratory physiology, applied sciences, and technological development have so far not been accompanied by a proportional increase in the development and diffusion of respiratory monitoring services. Here we show how fruitful synergies between different disciplines may provide avenues to address this issue (see Figure 2 for a schematic representation).

**Figure 3:**
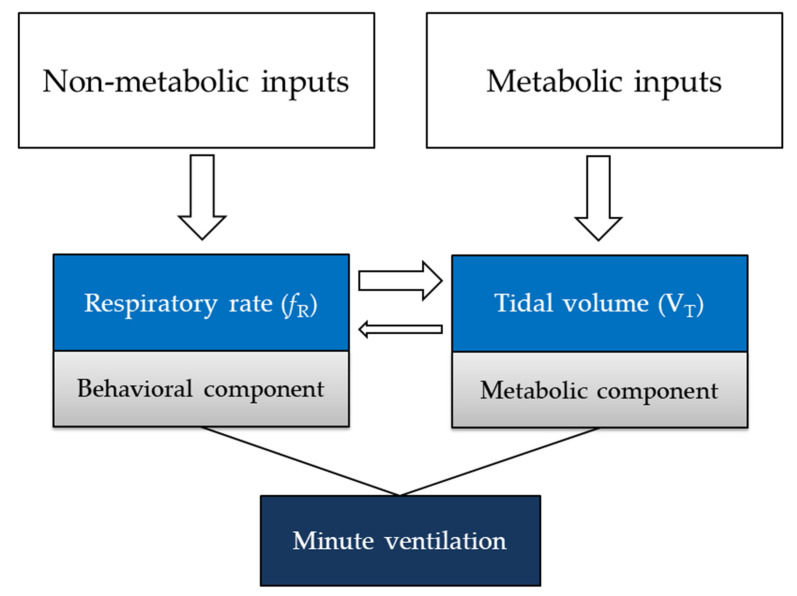
Schematic representation of a simple model of ventilatory control (see Nicolò and Sacchetti [22] for further information). While respiratory rate (the behavioral component of minute ventilation) is substantially influenced by non-metabolic stressors, V_T_ (the metabolic component of minute ventilation) satisfies the metabolic requirements of the human body. As such V_T_ is fine-tuned according to the levels of respiratory rate and the magnitude of metabolic inputs, while *f*_R_ is influenced by V_T_ to a lesser extent. This model explains why *f*_R_ is more sensitive than V_T_ to a variety of non-metabolic stressors and corroborates the importance of *f*_R_ monitoring in different fields of use.

Research in the field of respiratory physiology provides important insight into the rationale behind the choice of the ventilatory variables to monitor in different fields and applications. Indeed, the fact that *f*_R_ is more sensitive than tidal volume to a variety of stressors (see Section 2 for details) is in line with our current understanding of the control of ventilation. Increasing evidence suggests that *f*_R_ is substantially regulated by non-metabolic inputs [7,11,12,17,19,20,21,22], including brain areas relating to motor control, and those involved in emotion and cognitive processing [6,8,11,12,22]. This explains why *f*_R_ often increases in proportion to the extent of emotional stress, cognitive load, dyspnea, heat stimuli, and physical effort [7,8,9,12,13,18,22,210]. As such, *f*_R_ is the behavioral component of minute ventilation [19,20,22]. Conversely, most of these factors/conditions do not determine a consistent change in V_T_, which may even show opposite responses compared to those of *f*_R_, as observed under the influence of emotional and cognitive stimuli [6,8,193]. Indeed, V_T_ is the metabolic component of minute ventilation, and is adjusted on the basis of metabolic inputs and *f*_R_ levels to match alveolar ventilation with metabolic requirements [11,20,22]. In turn, *f*_R_ is influenced by V_T_, but to a minor extent compared to how V_T_ is affected by *f*_R_ [11,22]. These are the essential features (see Figure 3) of a recently-developed model of ventilatory control [11,12,19,22], which provides a physiological rationale for choosing when to monitor V_T_ alongside *f*_R_. The model shows that the measure of V_T_ is essential when respiratory monitoring is performed to identify the human response to metabolic stimuli like hypercapnia and metabolic acidosis. While this model deliberately simplifies the complexity of breathing control [7,19,20,22], it offers valuable insight on how to choose the ventilatory variables needed in different monitoring services.

Advances in the field of respiratory physiology are, in turn, favored by technological development and experimental evidence provided by applied sciences. For instance, the vast amount of evidence supporting the clinical relevance of *f*_R_ for a variety of diseases should guide basic research in the attempt to unravel the mechanisms underlying the commonly observed tachypneic breathing pattern. For instance, the well-documented importance of *f*_R_ in the context of cardiac arrest and severe pneumonia should trigger further research aiming to understand why *f*_R_ is particularly sensitive to these diseases. The importance of technological development for respiratory physiology and applied sciences is straightforward, as the widespread availability of accurate respiratory devices may speed up basic and applied research in different fields. This, in turn, would produce further knowledge to guide the development of respiratory measurement systems and monitoring services.

**Figure 4:**
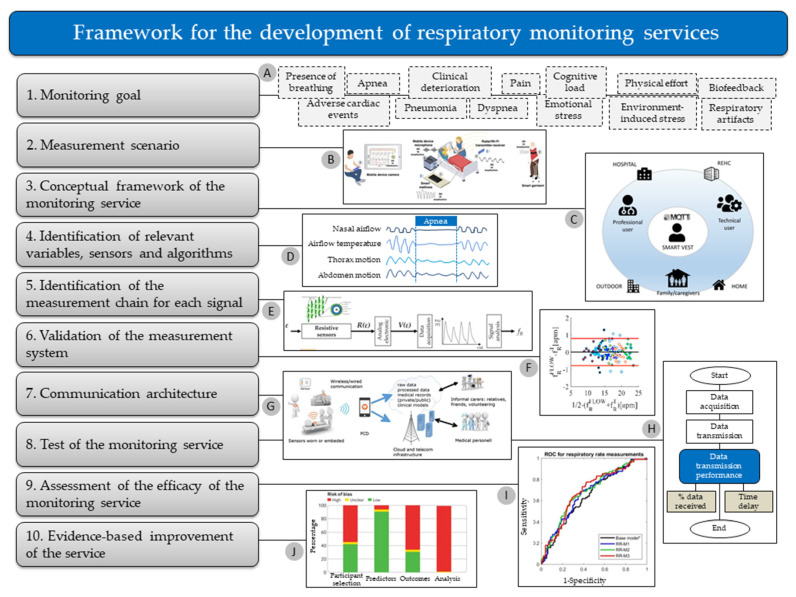
A conceptual framework for the development of respiratory monitoring services. The framework is composed of ten steps that are numbered and listed on the left-hand side of the figure. Each of the ten steps is accompanied by a graphical example reported on the right-hand side of the figure. Panel (**A**) reports the thirteen monitoring goals described in this review. The graph in panel (**B**) is reproduced from Massaroni et al. [5]. The graph in panel (**C**) is reproduced from Naranjo-Hernández et al. [157]. Panel (**D**) provides an example of the output of some of the sensors used to detect apnea events in sleep laboratories. The graph in panel (**E**) is slightly modified from Massaroni et al. [24]. The graph in panel (**F**) is slightly modified from Lo Presti et al. [290]. The graph in panel (**G**) is reproduced from Tomasic et al. [293]. Panel (**H**) provides an example of data transmission performance evaluation. The graph in panel (**I**) is slightly modified from Quinten et al. [116]. The graph in panel (**J**) is slightly modified from Gerry et al. [110].

The need for a multidisciplinary approach to respiratory monitoring is also manifested by the different levels of expertise required to structure respiratory monitoring services. As shown in Section 2, the development of new technologies should be guided by specific monitoring needs because measurement requirements depend on monitoring goals and measurement scenarios. Yet, respiratory devices are often not developed for specific purposes. An example is the limited diffusion of respiratory wearables specifically designed for monitoring sporting activities [14], where different measuring challenges arise. Likewise, limitations are encountered in the development of respiratory devices to support the diagnosis of pneumonia, especially in low-resource settings, where specific requirements are needed [89]. To partially address these challenges, we have developed a conceptual framework that may help to guide the development of respiratory monitoring services (see Figure 4).

The definition of monitoring goals is the first step in the development of a respiratory monitoring service. This is fundamental because the technological solutions identified may change extensively if the monitoring goal is, for instance, to detect apnea, identify emotional stress, or measure physical effort during exercise. It is also important to define the specific measurement scenario, which should orient the choice of measurement techniques. An example is provided in Figure 4 (panel B), which reports different measurement techniques for the remote respiratory monitoring of COVID-19 patients, depending on the need for periodic vital sign monitoring, or continuous monitoring [5]. Several other examples are provided in Section 2. When the monitoring goal and scenario are established, the development of a conceptual framework of the monitoring service will help identify relevant characteristics of the service, including users, resources, and facilities. An example is the framework proposed by Naranjo-Hernández et al. [157] (Figure 4, panel C), where a smart garment and a communication platform enable the remote monitoring of COPD patients by healthcare professionals and caregivers in different scenarios (i.e., hospital, e-health center, home and outdoor monitoring).

The next step is the identification of relevant variables, sensors and algorithms. The above considerations on when to monitor V_T_ provide an example of how to choose relevant ventilatory variables. Similar considerations may lead to the identification of other physiological and mechanical variables in order to achieve the desired goals. The different variables selected may be computed from different signals (e.g., respiratory waveform, ECG, and accelerometer signal), each requiring the use of specific sensors and algorithms. The different signal outputs can be combined to obtain monitoring features and metrics. An example is the detection of apnea/hypopnea events, which is often performed with the simultaneous recording of signals coming from different sensors (see Figure 4, panel D). For each signal selected, the elements composing the measurement chain need to be identified, i.e., measurand, sensor, electronics, and data acquisition, and signal analysis stages. As detailed by Massaroni et al. [24], the elements of the measurement chain change according to the different sensors used, and an example is provided in Figure 4 (panel E) for resistive sensors used to measure *f*_R_.

The output of all the signals should be compared with similar signals coming from reference systems, and each variable of interest should be validated. The validation output is affected by different factors, including the choice of the reference system, the algorithms used for signal processing, the validation indices selected, and the validation protocol and scenario. While it is outside of the scope of the present manuscript to discuss these points, the interested reader is referred to previous studies providing more details on this issue [26,62,65,89]. As an example, commonly computed validation indices are Mean of Difference and Limits of Agreement, which provide information on both the accuracy and precision of the tested measurement system. These indices are often depicted graphically by means of the Bland-Altman plot [289], as shown in the example provided in Figure 4 (panel F) [290].

Based on the characteristics of the measurement system and the respiratory service, a suitable communication architecture needs to be identified to enable the service. Three different layers (or tiers) can usually be identified in a communication architecture [291,292]. The first layer pertains to the communication between the body sensors and the sink, and is therefore called sensor-based tier [291,292]. The second layer is called gateway-based tier and pertains to the communication between the sink and one or multiple access points (e.g., smart device) [291,292]. The third layer pertains the communication beyond the access points and is usually composed of a medical server, a patient database, and a medical environment (in healthcare monitoring systems) [291,292]. Figure 4 (panel G) shows an example of a communication architecture used for the remote monitoring of COPD patients, where data from wearable sensors are acquired by a smart device through wireless communication and are streamed to health professionals and caregivers through cloud and telecom infrastructures [293].

Prior to service implementation in real contexts, a performance assessment of the service needs to be performed. Some of the factors that can be evaluated at this level are data transmission performance, data security, and user-friendliness [294,295,296]. Among different options, data transmission performance can be assessed with metrics like % data received and time delay [295], as reported in the example provided in Figure 4 (panel H). Once the service is implemented in routine activities, the efficacy of the service needs to be evaluated. The assessment methodology may depend on the specific service developed. A recent study [296] has reported an interesting methodological approach to evaluate the efficacy of smartphone-based respiratory monitoring services. The authors have identified four categories determining the efficacy of respiratory services, i.e., smartphone performance metrics factors (e.g., data security and privacy level), patient status factors (e.g., level of patient satisfaction), cost-related factors (e.g., cost to the customers), and resource-related factors (e.g., internet connectivity level) [296]. For the healthcare services based on the use of early warning scores to predict clinical outcomes, the efficacy is commonly evaluated by computing metrics of specificity and sensitivity, and by producing receiver operating characteristic (ROC) curves [110]. An example is provided in Figure 4 (panel I), which reports ROC curves for repeated respiratory rate measurements collected within the first hours from admission to an emergency department [116]. In this example, the ROC curves show that repeated measurements of respiratory rate are better associated with patient deterioration compared to a single measurement at hospital admission [116]. However, recent systematic reviews have found little evidence of any clinical effectiveness of the early warning scores commonly used in the clinical setting [110,297,298]. This finding can be largely attributed to a series of methodological weaknesses related to the development and validation of early warning scores, including participant selection, the choice of outcome measures, and the analysis performed (Figure 4, panel J) [110]. To overcome this problem, Gerry et al. [110] have provided a series of recommendations on population description, sample size, missing data management, outcome measures, time horizons, statistical methods, validation methodology, and on the selection of metrics for testing model performance [110]. This is an emblematic example of how evidence-based approaches should guide the improvement of respiratory monitoring services. Given the ever-increasing growth of science and technology in the field of respiratory monitoring, an evidence-based approach should also be used to improve respiratory monitoring services at any step of the here proposed framework.

## 4. Perspectives and Challenges of Respiratory Rate Monitoring

**Figure 5:**
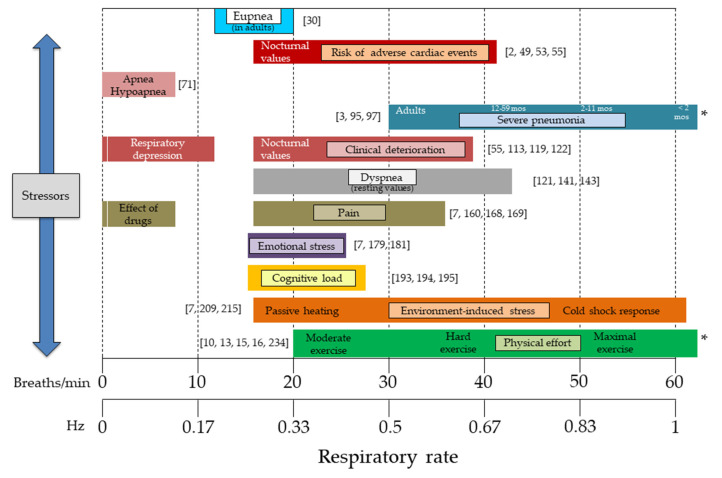
Schematic representation of how respiratory rate (values expressed both in breaths/min and in Hz) may change in response to different stressors. The range of respiratory rate values reported for each stressor has been defined according to the cited references (numbers in square brackets), but these values should only be considered as plausible examples. *f*_R_ values refer to adults if not otherwise stated. * it is not unusual to observe *f*_R_ values higher than 65 breaths/min. Mos, months.

Despite the importance of *f*_R_ in different fields of use, the fact that *f*_R_ is sensitive to a variety of non-metabolic stressors [7,8,9,11,12,19,20,21] may impair our understanding of the factors determining an increase in *f*_R_. As shown in Figure 5, similar values of *f*_R_ can be observed when the user is under cognitive load, emotional stress, pain, dyspnea, or is simply performing moderate exercise. This problem can be partly overcome with the simultaneous measurement of other physiological and mechanical variables. For instance, it is important to characterize the postures and activities of the user, as commonly performed with the use of inertial sensors [299]. This is particularly relevant for monitoring dyspnea and physical effort during everyday life activities and exercise [27], but is also important for the identification of suitable portions of the respiratory signal (e.g., with no movements or artifacts) to compute resting *f*_R_ [133]. The concomitant measure of other vital signs is also beneficial, as in the development of early warning scores for the prediction of clinical deterioration [4]. Besides, *f*_R_ and its variability change across wakefulness and different sleep stages [300,301], which is relevant when interpreting nocturnal *f*_R_ values. Sleep stages are usually identified with electroencephalography, but approaches based on breathing sound processing have also been proposed [81]. Another strategy to gain insight into the factors behind the changes in *f*_R_ is the recording of concomitant symptoms, including pain, dyspnea, and emotional stress. These symptoms can be assessed with validated scales [302,303], but this approach can only be used on collaborative patients.

Computing solutions may also provide insight into the factors affecting *f*_R_. For instance, analyses of ventilatory variability have been used to identify cognitive load and emotional stress [9,191], but further research is needed to guide their implementation in respiratory monitoring services. Artificial intelligence approaches can also be applied to respiratory monitoring for the identification of the different stressors reported in Figure 5. Some of these methods of analysis have been used in the field of apnea monitoring [87], for the prediction of clinical deterioration in COVID-19 patients [304], and for the identification of opioid-induced ataxic breathing [173]. Another common issue in the field of healthcare is the use of fixed (sometimes arbitrary) cut-off values of *f*_R_ [100,119], which do not take into account inter-individual differences in *f*_R_ resting values and in the responses to different stressors [3,305]. Most of these challenges can be overcome with the development of accurate respiratory systems and the effective implementation of respiratory monitoring services in routine use. This would lead to the availability of a large amount of respiratory data obtained in different measurement scenarios, hence fostering the processes described in Figure 2 and Figure 4.

## 5. Conclusions

This review presents a multidisciplinary approach to respiratory rate monitoring, with the aim to improve the development of respiratory monitoring services in different fields of use. We have identified thirteen monitoring goals where the measure of respiratory rate is invaluable and presented suitable *f*_R_ measurement techniques for specific measurement scenarios. The variety of monitoring goals presented has allowed us to show differences in the state of development of respiratory services across different fields of use. For instance, the field of apnea detection is regulated by detailed guidelines on the use of respiratory sensors, while the field of pneumonia suffers from a lack of consensus on how to accurately and objectively measure *f*_R_. Likewise, respiratory monitoring requires more consideration and development in the field of sport and exercise, where *f*_R_ is emerging as a valid marker of physical effort and exercise-induced fatigue. We hope that the multidisciplinary approach presented may have contributed to corroborate the importance of measuring *f*_R_ in different fields, and to provide solutions for the effective development of respiratory monitoring services.

## Figures and Tables

**Table 1:** Summary of measurement guidelines for each monitoring goal identified.

Monitoring Goal	Contact-Based Methods	Contactless Methods	Information Detail	Type of Recording	Main Measurement/Computing Challenge	Need for V_T_ *
1. Presence of breathing	XXX	XX	b-by-b	P/C	respiratory signal quality	-
2. Adverse cardiac events	XXX	X	60 s	C	wearable and unobtrusive systems	-
3. Apnea	XXX	X	raw data	C	hypopnea detection	●●●
4. Pneumonia	XXX	XX	60 s	P/C	solutions for low-income countries	-
5. Clinical deterioration	XXX	XX	60 s	P/C	acceptance of technologies	-
6. Dyspnea	XXX	X	b-by-b	C	motion artifacts	●●
7. Pain	XXX	X	b-by-b/60s	C	detection of respiratory depression	●●
8. Emotional stress	XXX	XX	b-by-b	P/C	processing of video images	●
9. Cognitive load	XXX	XX	b-by-b/60 s	C	accurate and unobtrusive systems	-
10. Environment-induced stress	XXX	X	60 s	C	change of sensor properties	●
11. Physical effort	XXX	X	b-by-b/5 s	C	motion artifacts	-
12. Respiratory artifacts	XX	XXX	raw data	P	respiratory features in real-time	●●●
13. Respiratory biofeedback	XXX	XX	raw data	P	respiratory features in real-time	●●

X, can be used in some instances; XX, suitable; XXX, advised solution; b-by-b, breath-by-breath; P, periodic monitoring; C, continuous monitoring; V_T_, tidal volume; *, or respiratory amplitude as a surrogate for tidal volume; -, not necessarily needed; ●, useful; ●●, very useful; ●●●, required.

## Abbreviations

The manuscript contains the following abbreviations:

AHI	Apnea-Hypopnea Index
COPD	Chronic obstructive pulmonary disease
CSA	Central sleep apnea
CT	Computed Tomography
ECG	Electrocardiography
*f*_R_	Respiratory frequency
ICU	Intensive care unit
LOAs	Limits of agreement
MEWS	Modified Early Warning Score
MOD	Mean of difference
mos	Months
MRI	Magnetic Resonance Imaging
NEWS	National Early Warning Score
OSA	Obstructive sleep apnea
PET	Positron Emission Tomography
PPG	Photoplethysmography
RGB	Red green blue
RIP	Respiratory inductive plethysmography
ROC	Receiver operating characteristic
SQI	Signal quality index
UWB	Ultra-wideband
V_T_	Tidal volume
